# Cell wall-localized BETA-XYLOSIDASE4 contributes to immunity of Arabidopsis against *Botrytis cinerea*

**DOI:** 10.1093/plphys/kiac165

**Published:** 2022-04-18

**Authors:** Athanas Guzha, Robert McGee, Patricia Scholz, Denise Hartken, Daniel Lüdke, Kornelia Bauer, Marion Wenig, Krzysztof Zienkiewicz, Cornelia Herrfurth, Ivo Feussner, A Corina Vlot, Marcel Wiermer, George Haughn, Till Ischebeck

**Affiliations:** Department of Plant Biochemistry, Albrecht-von-Haller-Institute for Plant Sciences and Goettingen Center for Molecular Biosciences (GZMB), University of Goettingen, Justus-von-Liebig Weg 11, D-37077 Goettingen, Germany; Department of Botany, University of British Columbia, Vancouver, British Columbia, Canada V6T 1Z4; Department of Plant Biochemistry, Albrecht-von-Haller-Institute for Plant Sciences and Goettingen Center for Molecular Biosciences (GZMB), University of Goettingen, Justus-von-Liebig Weg 11, D-37077 Goettingen, Germany; Molecular Biology of Plant-Microbe Interactions Research Group, Albrecht-von-Haller-Institute for Plant Sciences and Goettingen Center for Molecular Biosciences (GZMB), University of Goettingen, Justus-von-Liebig Weg 11, D-37077 Goettingen Germany; Molecular Biology of Plant-Microbe Interactions Research Group, Albrecht-von-Haller-Institute for Plant Sciences and Goettingen Center for Molecular Biosciences (GZMB), University of Goettingen, Justus-von-Liebig Weg 11, D-37077 Goettingen Germany; Helmholtz Zentrum Muenchen, Institute of Biochemical Plant Pathology, Ingolstaedter Landstrasse 1, 85764 Neuherberg, Germany; Helmholtz Zentrum Muenchen, Institute of Biochemical Plant Pathology, Ingolstaedter Landstrasse 1, 85764 Neuherberg, Germany; Department of Plant Biochemistry, Albrecht-von-Haller-Institute for Plant Sciences and Goettingen Center for Molecular Biosciences (GZMB), University of Goettingen, Justus-von-Liebig Weg 11, D-37077 Goettingen, Germany; Service Unit for Metabolomics and Lipidomics, Goettingen Center for Molecular Biosciences (GZMB), University of Goettingen, D-37077 Goettingen, Germany; UMK Centre for Modern Interdisciplinary Technologies, Nicolaus Copernicus University, 87-100 Toruń, Poland; Department of Plant Biochemistry, Albrecht-von-Haller-Institute for Plant Sciences and Goettingen Center for Molecular Biosciences (GZMB), University of Goettingen, Justus-von-Liebig Weg 11, D-37077 Goettingen, Germany; Service Unit for Metabolomics and Lipidomics, Goettingen Center for Molecular Biosciences (GZMB), University of Goettingen, D-37077 Goettingen, Germany; Department of Plant Biochemistry, Albrecht-von-Haller-Institute for Plant Sciences and Goettingen Center for Molecular Biosciences (GZMB), University of Goettingen, Justus-von-Liebig Weg 11, D-37077 Goettingen, Germany; Service Unit for Metabolomics and Lipidomics, Goettingen Center for Molecular Biosciences (GZMB), University of Goettingen, D-37077 Goettingen, Germany; Helmholtz Zentrum Muenchen, Institute of Biochemical Plant Pathology, Ingolstaedter Landstrasse 1, 85764 Neuherberg, Germany; Molecular Biology of Plant-Microbe Interactions Research Group, Albrecht-von-Haller-Institute for Plant Sciences and Goettingen Center for Molecular Biosciences (GZMB), University of Goettingen, Justus-von-Liebig Weg 11, D-37077 Goettingen Germany; Freie Universität Berlin, Institute of Biology, Dahlem Centre of Plant Sciences, Biochemistry of Plant-Microbe Interactions, Königin-Luise-Str. 12-16, 14195 Berlin, Germany; Department of Botany, University of British Columbia, Vancouver, British Columbia, Canada V6T 1Z4; Department of Plant Biochemistry, Albrecht-von-Haller-Institute for Plant Sciences and Goettingen Center for Molecular Biosciences (GZMB), University of Goettingen, Justus-von-Liebig Weg 11, D-37077 Goettingen, Germany; Institute of Plant Biology and Biotechnology (IBBP), Green Biotechnology, University of Münster, Schlossplatz 8, D-48143 Münster, Germany

## Abstract

Plant cell walls constitute physical barriers that restrict access of microbial pathogens to the contents of plant cells. The primary cell wall of multicellular plants predominantly consists of cellulose, hemicellulose, and pectin, and its composition can change upon stress. *BETA-XYLOSIDASE4* (*BXL4*) belongs to a seven-member gene family in Arabidopsis (*Arabidopsis thaliana*), one of which encodes a protein (*BXL1*) involved in cell wall remodeling. We assayed the influence of BXL4 on plant immunity and investigated the subcellular localization and enzymatic activity of BXL4, making use of mutant and overexpression lines. BXL4 localized to the apoplast and was induced upon infection with the necrotrophic fungal pathogen *Botrytis cinerea* in a jasmonoyl isoleucine-dependent manner. The *bxl4* mutants showed a reduced resistance to *B. cinerea*, while resistance was increased in conditional overexpression lines. Ectopic expression of *BXL4* in Arabidopsis seed coat epidermal cells rescued a *bxl1* mutant phenotype, suggesting that, like BXL1, BXL4 has both xylosidase and arabinosidase activity. We conclude that BXL4 is a xylosidase/arabinosidase that is secreted to the apoplast and its expression is upregulated under pathogen attack, contributing to immunity against *B. cinerea*, possibly by removal of arabinose and xylose side-chains of polysaccharides in the primary cell wall.

## Teaser

A putative bifunctional xylosidase/arabinofuranisodase localizes to the apoplast and is important for immunity against the necrotrophic pathogen *Botrytis cinerea*.

## Introduction

Plants are continuously exposed to a plethora of biotic threats such as herbivorous insects and microbial pathogens. To help mitigate these threats, plants have evolved various inducible and constitutive defense mechanisms against biotic and abiotic perturbations (Jones and Dangl, 2006; Dangl et al., 2013). Induced immune responses are diverse and include the production of various phytohormones that are activated by different classes of microbial pathogens, insect pests, and abiotic stresses (McDowell and Dangl, 2000; Lebeis et al., 2015). Important constitutive barriers that plant pathogens must overcome to access cellular contents are the cuticle and the plant cell wall (Vorwerk et al., 2004; Underwood, 2012; Engelsdorf et al., 2017; Escudero et al., 2017). The importance of cell walls for plant defense is demonstrated by the abundance of cell wall degrading enzymes that microbial pathogens secrete in order to successfully invade plant tissues (Glass et al., 2013; Quoc and Bao Chau, 2017; Hao et al., 2019). The plant immune system in turn reacts to cell wall degradation products such as oligogalacturonides (OGs; Ferrari, 2013; Davidsson et al., 2017), hemicellulose derived β-1,3-1,4-glucan oligosaccharides (Barghahn et al., 2021; Yang et al., 2021), arabinoxylan oligosaccharides (Mélida et al., 2020), and cellulose derived oligosaccharides (Zarattini et al., 2021). Plant cell walls consist of a complex meshwork of polysaccharides, where cellulose microfibrils are cross-linked by various hemicelluloses and embedded in a pectic matrix. Cellulose consists of (1-4)-β-linked d-glucose residues and is synthesized by cellulose synthase complexes located in the plasma membrane (Somerville, 2006; Carpita, 2011). Hemicelluloses are a diverse group of polysaccharides. In Arabidopsis (*Arabidopsis thaliana*), the most abundant hemicellulose is xyloglucan, which is characterized by a (1,4)-β-linked glucan regularly substituted with (1-6)-α-xylosyl residues (Zablackis et al., 1995; Scheller and Ulvskov, 2010; Pauly and Keegstra, 2016; Höfte and Voxeur, 2017).

Pectin is the most complex cell wall polysaccharide and its biosynthesis involves at least 67 different enzyme activities (Harholt et al., 2010). Pectin consists of four types of polysaccharides: homogalacturonan (HG), xylogalacturonan, and rhamnogalacturonan (RG) I and II (Mohnen, 2008). HG, xylogalacturonan, and RG-II are all characterized by the presence of a (1-4)-α-d-galacturonic acid backbone, whereas RG-I, has a backbone alternating in (1-2)-α-l-rhamnose and (1-4)-α-d-galacturonic acid residues (O’Neill et al., 1990; Ridley et al., 2001; Mohnen, 2008; Mohnen et al., 2008). RG-I is also characterized by arabinan, galactan, and arabinogalactan side chains, and xylan side chains were proposed to exist as well (Ralet et al., 2016). In Arabidopsis, pectin is most abundant in the primary cell walls (Zablackis et al., 1995) and it is important for the regulation of cell wall mechanical properties during growth and development (Moore et al., 2008). It also influences water imbibition of seeds, pollen tube growth, leaf and flower abscission, fruit ripening, and cell wall integrity induced signaling (Mohnen, 2008; Arsovski et al., 2009; Harholt et al., 2010; Kohorn and Kohorn, 2012).

In addition to polysaccharides, plant cell walls also contain various proteins (Albenne et al., 2014). These proteins are an integral part of the cell wall, as they contribute to its structural integrity, or modify cell wall composition during plant development and in response to environmental cues (Fry, 2004; Passardi et al., 2004). One major group of cell wall modifying proteins are pectin methylesterases (PMEs), which demethylesterify the pectin HG after its biosynthesis (Lionetti et al., 2014). At least 67 PME isoforms are thought to be present in Arabidopsis (Lionetti et al., 2017). The activity of these PMEs is tightly regulated by PME inhibitor (PMEI) proteins which exist in families equally as large as the PMEs (Wormit and Usadel, 2018). HG is also modified by polygalacturonases that hydrolyse the glycosidic linkages of the galacturonic acid backbone (Xiao et al., 2014).

The modifications occurring on pectin such as demethylesterification and acetylation (Liu et al., 2018) are known to play a role in cell wall integrity maintenance and resistance to pathogens. For example, Arabidopsis *reduced wall acetylation 2* mutants, that have reduced pectin acetylation, are more resistant to *Botrytis cinerea* (Manabe et al., 2011). The alteration of HG has also been shown to enhance resistance to *B. cinerea* (Lorrai et al., 2021). Arabidopsis plants overexpressing *PMEI-1* and *PMEI-2* show enhanced resistance to the phytopathogens *B. cinerea* and *Pectobacterium carotovorum* (Lionetti et al., 2007). Another pectin modification important for pathogen defense is the oxidation of OGs derived from HG hydrolysis (Benedetti et al., 2018). In Arabidopsis, four members of a berberine bridge enzyme-like family were found to be responsible for this oxidation. Although the oxidized OGs trigger weaker immune responses, they are more resistant to hydrolysis by *B. cinerea* enzymes. Accordingly, Arabidopsis plants overexpressing these oxidases are less susceptible to this pathogen (Benedetti et al., 2018).

As highlighted above, many defense mechanisms involving pectin are attributed to HG and its methylation status. However, several questions still exist about how the minor pectins such as RG-I and RG-II together with their modification affect plant–pathogen interactions. In this study, we investigated the role of the Arabidopsis protein BETA-D-XYLOSIDASE4 (BXL4, AtBXL4, and XYL4) and its impact on plant immunity. All seven Arabidopsis BXL family members (BXL1-BXL7) possess predicted glycosyl hydrolase domains, whilst some have predicted signal peptides for extracellular localization (Goujon et al., 2003). Only BXL1 has been studied in detail. It was shown to be a bifunctional β-D-xylosidase/α-L-arabinofuranosidase (Goujon et al., 2003; Minic et al., 2004; Arsovski et al., 2009) that is important for extrusion of pectin rich mucilage upon hydration of Arabidopsis seeds (Arsovski et al., 2009) as it removes the side chains present in RG-I (Williams et al., 2020) and is associated with tissues undergoing secondary cell wall thickening (Goujon et al., 2003). We provide evidence that another member of this protein family, BXL4, localizes to the plant cell wall, where it most likely acts on both xylans and arabinans. We show that *BXL4* expression is induced by *B. cinerea* infection and by mechanical wounding in a jasmonoyl-isoleucine (JA-Ile) dependent manner. Accordingly, *bxl4* mutants are more susceptible when challenged with *B. cinerea* and display reduced levels of JA-Ile and camalexin after infection suggesting that BXL4 induction is part of the defense response. Consistent with this hypothesis, overexpression of *BXL4* results in increased transcript accumulation of the *B. cinerea*-responsive marker genes *PLANT DEFENSIN 1.2* (*PDF1.2*) and *PHYTOALEXIN DEFICIENT 3* (*PAD3*) and enhanced resistance to *B. cinerea*. Taken together, our data provide evidence of an important role for BXL4 in plant immunity.

## Results

### *BXL4* expression is induced by wounding and *B. cinerea* infection

**Figure 1:**
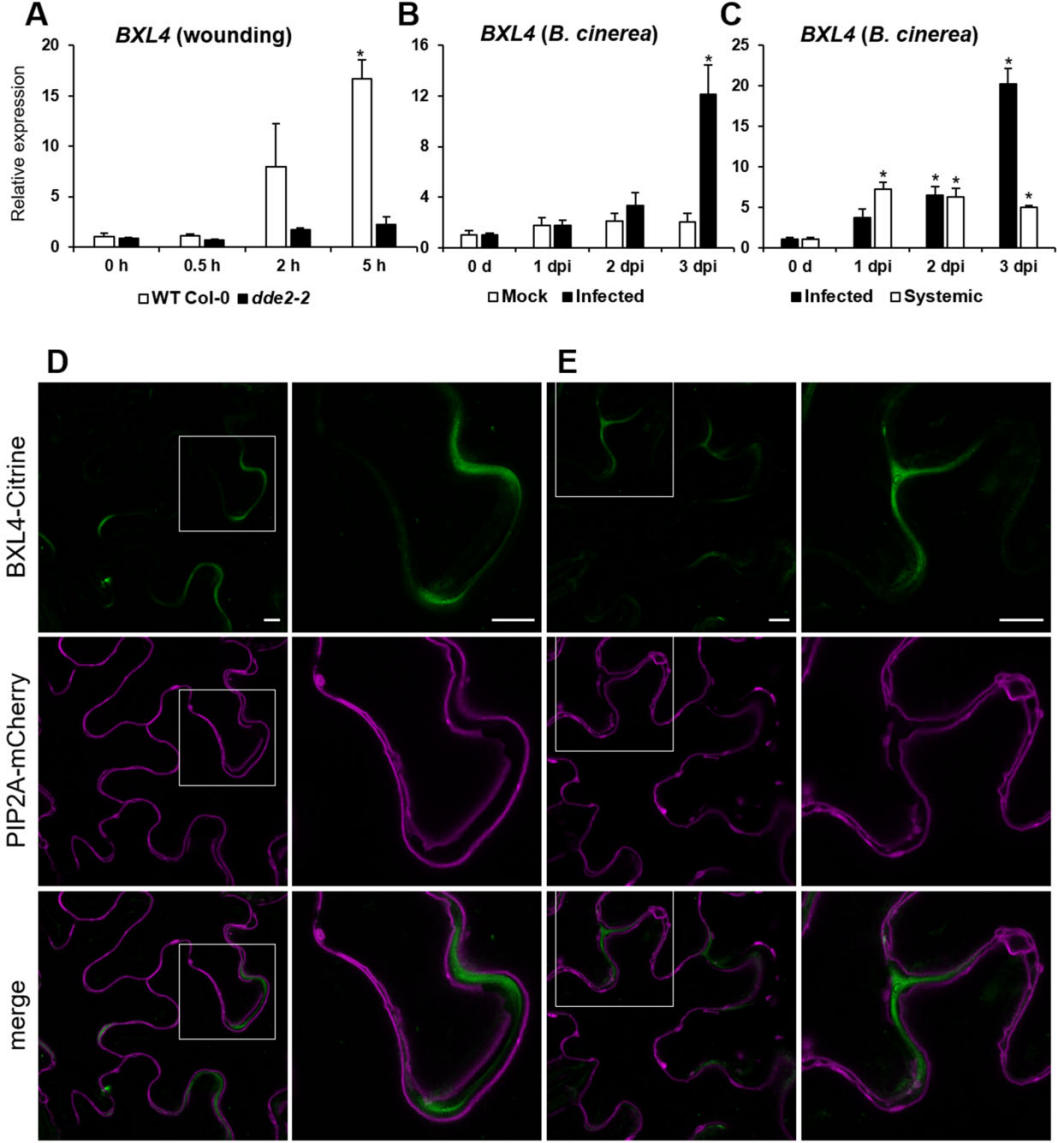
BXL4 is secreted to the apoplast and induced upon mechanical wounding and *B. cinerea* infection. A, Expression pattern of *BXL4* in Col-0 and the JA-Ile-deficient mutant *dde2-2.* RNA extracted from 6-week-old plants before wounding (0 h) and at 0.5-, 2-, and 5-h post wounding. B, Relative expression of *BXL4* in leaves of 6-week-old Col-0 plants after leaves were drop inoculated with *B. cinerea* conidiospores suspension or Vogel’s buffer (mock). C, Relative expression of *BXL4* in infected or unchallenged systemic (distal) leaves at 0 d (before inoculation) and 1, 2, and 3 dpi. Error bars in (A), (B), and (C) show se (*n* = 3 biological replicates each performed on RNA extracted from whole individual plants for A and B, or from four infected leaves or four noninfected leaves from individual plants for C); statistical differences to the WT at 0 h (A) and 0 days (B and C) were determined (Student’s *t* test; * indicates *P* < 0.05). Experiments were conducted three times with similar results. D, The BXL4-CITRINE fusion protein (green) localizes to the cell wall in *N. benthamiana* leaves. The plasma membrane marker PIP2A-mCherry (magenta) was also coexpressed in *N. benthamiana* leaves. Overlay of the images shows that the plasma membrane of adjoining cells is separated by the BXL4-CITRINE fluorescence. E, BXL4-CITRINE green fluorescence does not show gaps in the corners of adjoining cells which is consistent with cell wall localization. Images are representative for 55 images from nine leaves derived from four independent transformations. Bars = 10 µm.

BXL4 is part of a seven-member protein family of putative xylosidases in Arabidopsis (Supplemental Figure S1; Goujon et al., 2003), and the expression of *BXL4* is upregulated by infection with various pathogens according to publicly available databases (Winter et al., 2007; Hruz et al., 2008). To confirm that *BXL4* is a stress-induced gene, its expression pattern after mechanical wounding and infection with *B. cinerea* was analyzed (Figure 1, A–C). Generally, *BXL4* gene expression is relatively low in Columbia-0 (Col-0) grown under normal conditions (Supplemental Figure S2). However, the *BXL4* gene expression was induced 16-fold upon mechanical wounding of the rosettes of Col-0 (Figure 1A). *BXL4* expression was also investigated in the JA-Ile deficient mutant line *delayed-dehiscence2-2* (*dde2-2*; von Malek et al., 2002), because JA-Ile regulates the expression of many wounding responsive genes (Howe et al., 2018). Relative to wild-type (WT) expression, upregulation of *BXL4* transcript levels after wounding was greatly reduced in the *dde2-2* mutant with a two-fold induction at 5 h after wounding compared with the 16-fold induction in the WT (Figure 1A). Because Arabidopsis defense against necrotrophic pathogens is associated with JA-Ile (Pieterse et al., 2012; Zhang et al., 2017), accumulation of *BXL4* transcript after infection with *B. cinerea* was quantified. There was a significant 12- to 20-fold induction of *BXL4* transcript accumulation in Arabidopsis 3 days post inoculation (dpi) with *B. cinerea* (Figure 1B). To investigate whether the induction of *BXL4* expression after *B. cinerea* infection also occurs in unchallenged systemic tissues, the *BXL4* expression was analyzed in distal leaves after a local *B. cinerea* drop-inoculation. This analysis revealed that the *BXL4* expression is significantly induced in the uninfected distal leaves after local *B. cinerea* infection (Figure 1C).

### BXL4 localizes to the apoplast

Cell wall modification in muro is one important aspect of plant defense (Ferrari et al., 2012; Lionetti et al., 2017). The Arabidopsis protein BXL4 possesses a predicted signal peptide for secretion (Goujon et al., 2003) and showed increased abundance in the Arabidopsis apoplast after *Pseudomonas syringae* and *B. cinerea* infection (Breitenbach et al., 2014; Sham et al., 2014). To confirm cell wall localization, we transiently expressed BXL4-CITRINE under the control of the 35S promoter in *Nicotiana benthamiana* leaf epidermal cells together with the plasma membrane marker PLASMA MEMBRANE INTRINSIC PROTEIN 2A-mCHERRY (PIP2A-mCHERRY; Nelson et al., 2007). The results indicate that BXL4-CITRINE localizes to the apoplast between the plasma membranes of adjacent cells (Figure 1, D and E).

### Disruption of *BXL4* results in subtle modification of pectin composition but does not affect plant growth

**Figure 2:**
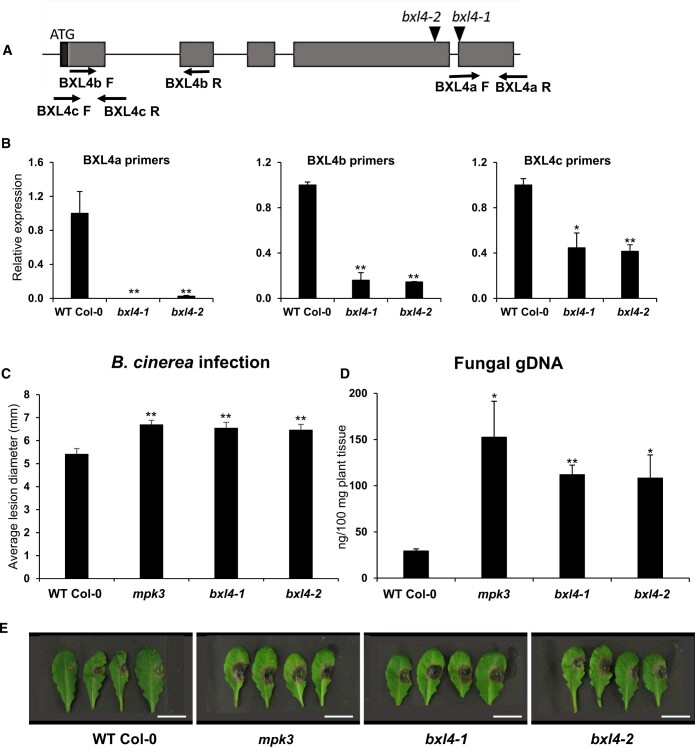
*bxl4* mutant lines are more susceptible to *B. cinerea* infection. A, The intron–exon structure of *BXL4* and the positions of T-DNA insertions in *bxl4-1* and *bxl4-2.* The exons are represented by gray boxes, introns by black lines, black triangles show positions of the T-DNA insertions that were confirmed by sequencing. The 5′ UTR is represented by a black box. Arrows indicate primers used for RT-qPCR analysis. B, Relative expression of *BXL4* in leaves of 4-week-old WT and *bxl4* mutant plants as determined by RT-qPCR using primers as indicated and shown in Supplemental Table S1. Expression values were normalized to *ACTIN8* and are shown relative to levels in Col-0. Error bars show se (*n* = 3 biological replicates each performed on RNA extracted from whole individual plants), statistical differences to the WT were determined (Student’s *t* test, * indicates *P* < 0.05; **P < 0.01). C, Infection phenotype of Col-0, *bxl4-1*, and *bxl4-2* after *B. cinerea* infection. A minimum of 30 leaves from 5 independent plants were drop inoculated with 6 µL *B. cinerea* conidiospores, and lesion diameter measured with a digital caliper 3 dpi. *mpk3* was used as the susceptible control. Error bars show se (*n* ≥ 30 leaves), statistical differences to the WT were determined (Student’s *t* test, ** indicates *P* < 0.01). Experiment was conducted four times with similar results. D, Infection phenotype measured after spraying plants with *B. cinerea* conidiospores and quantifying fungal genomic DNA by qPCR. Fungal genomic DNA was quantified 3 dpi. Error bars represent se (*n *= 3 biological replicates each performed on RNA extracted from whole individual plants), statistical differences to the WT were determined (Student’s *t* test, * indicates *P* < 0.05; **P < 0.01). The experiment was conducted times times with similar results. E, Lesion phenotype of detached leaves of Col-0, *mpk3*, *bxl4-1*, and *bxl4-2* at 3 dpi with *B. cinerea*. Scale bar, 10 mm.

Alteration of the cell wall composition may impair normal growth and development of Arabidopsis plants (Noguchi et al., 1997). To test if the disruption of *BXL4* gene function affects the normal growth of Arabidopsis, two independent Arabidopsis T-DNA insertion lines, *bxl4-1* and *bxl4-2*, that carry insertions in exons 5 and 4, respectively, were obtained from the Nottingham Arabidopsis stock center (Figure 2A). The position of the T-DNA insertion was confirmed by sequencing and *BXL4* transcript levels were analyzed in the T-DNA insertion lines by reverse transcription-quantitative PCR (RT-qPCR) using primers that amplify regions of *BXL4* both upstream and downstream of the T-DNA insertion locus. Whereas we could not detect full-length *BXL4* transcripts using primers downstream of the T-DNA insertion in the *bxl4-1* line, *BXL4* transcript accumulation was detectable, but significantly reduced, in *bxl4-2* plants (Figure 2B). Use of primers upstream of the T-DNA insertion site revealed a significant reduction of *BXL4* transcript accumulation in the *bxl4* mutants compared with Col-0 (Figure 2B). To evaluate if the disruption of *BXL4* gene function in *bxl4-1* and *bxl4-2* affects the monosaccharide composition of pectin in unchallenged tissues, water-extracted pectin from alcohol insoluble residues (AIRs) of leaf material was analyzed for monosaccharide composition using a modified work-flow based on gas chromatography–mass spectrometry (GC–MS; Biswal et al., 2017). The monosaccharide compositions were normalized to the total sugars. The two *bxl4* mutant lines showed a slightly increased relative abundance of arabinose compared with Col-0 (Supplemental Figure S3A). However, there was no significant difference in xylose or other pectin monosaccharides measured of either line. Dot blot assays carried out on pectin extracted from the three genotypes indicate no changes in HG or RG I (Supplemental Figure S3, B and C) but increased abundance of long stretches of 1,5-linked arabinosyl residues, especially in the *bxl4-1* mutant (Supplemental Figure S3D). These residues are believed to be side chains of RG-I (Arsovski et al., 2009). Despite these subtle effects on leaf cell wall pectin composition, neither *bxl4* mutant exhibited any obvious growth defects and was comparable to Col-0 (Supplemental Figure S4).

### BXL4 contributes to resistance against *B. cinerea*

As the expression of *BXL4* is induced after *B. cinerea* infection (Figure 1), we tested if the *bxl4* mutants are compromised in resistance to *B. cinerea* by drop inoculating plants with *B. cinerea* conidiospore suspensions and measuring the lesion area 3 dpi (Figure 2, C and E). The *bxl4* mutants developed significantly larger lesions compared with Col-0. The *mpk3* mutant was used as a control with enhanced susceptibility (Galletti et al., 2011). An additional method was used to quantify the disease susceptibility to *B. cinerea*. Plants were sprayed with *B. cinerea* conidiospore suspension and the fungal β-ACTIN genomic DNA was quantified 3 days after spray inoculation using qPCR (Ettenauer et al., 2014). The *bxl4* mutants showed a significantly higher abundance of fungal genomic DNA when compared with Col-0 (Figure 2D).

**Figure 3:**
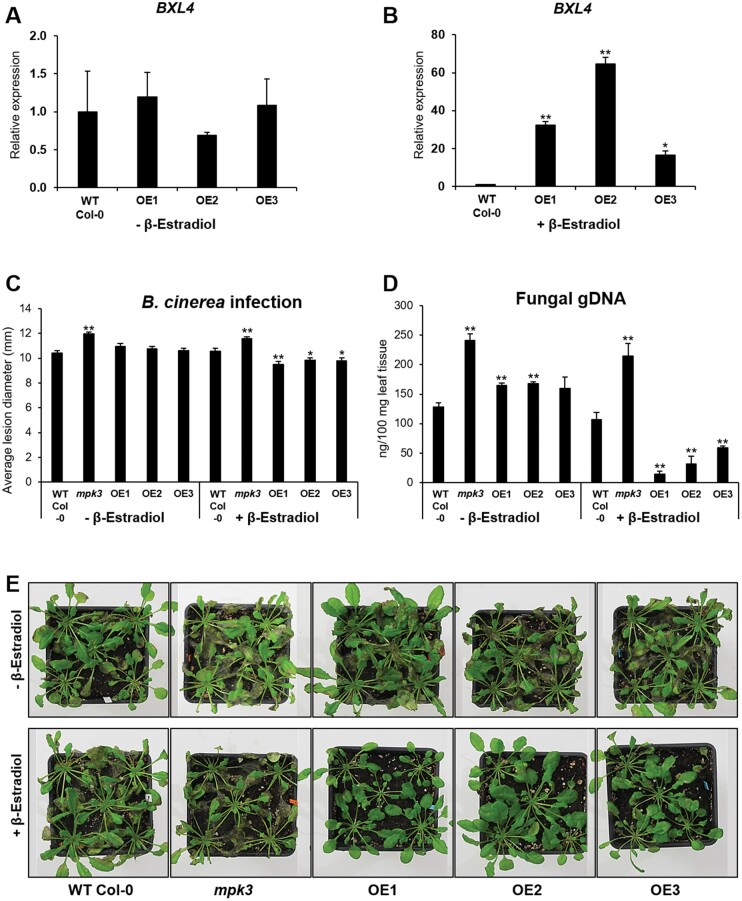
The overexpression of *BXL4* results in increased resistance to *B. cinerea.* The relative expression of *BXL4* at 4 days after mock induction (A) and after β-estradiol induction (B) of *BXL4* inducible overexpression lines 1, 2, and 3 (OE1, OE2, and OE3). C, Lesion diameter of Col-0, *mpk3*, and inducible overexpression lines 1, 2, and 3. Plants were treated with β-estradiol 4 days prior to infection with *B. cinerea* and lesion diameters were scored 3 dpi. Error bars show se (*n* ≥ 30 leaves from at least five plants), statistical differences to the WT were determined (Student’s *t* test, * indicates *P* < 0.05, **P < 0.01). D, quantification of fungal genomic DNA measured after spraying plants with *B. cinerea* conidiospores and quantifying with qPCR. Fungal genomic DNA was quantified 3 dpi. The overexpression of *BXL4* was either mock induced or continuously induced once per week for 6 weeks before spraying with *B. cinerea* conidiospores. Error bars represent sd (*n* = 3 biological replicates each performed on RNA extracted from whole individual plants), statistical differences to the WT were determined (Student’s *t* test ** indicates *P* < 0.01). E, infection phenotype of Arabidopsis plants after mock or β-estradiol induction: Col-0, *mpk3*, OE1, OE2, and OE3 at 3 dpi with *B. cinerea*. BXL4 overexpression was continuously induced.

To assess the effects of BXL4 overexpression on Arabidopsis defense reactions, three independent transgenic inducible overexpression lines (OE1, OE2, and OE3) containing a *BXL4* transgene under control of an estradiol-inducible promoter were generated in the Col-0 background. The expression of *BXL4* was evaluated by RT-qPCR in 6-week-old Arabidopsis plants 4 days after spraying with 0.01% Tween 20 (mock) or 50 µM β-estradiol (Figure 3, A and B). The mock-treated plants did not show any induction of *BXL4* expression (Figure 3A), but there was a 15- to 60-fold increase compared with Col-0 in the β-estradiol-induced lines (Figure 3B).

We then tested if the overexpression of *BXL4* also had an effect on immunity to *B. cinerea*. The inducible *BXL4* overexpression lines were drop inoculated with *B. cinerea* conidiospores 4 days after β-estradiol induction, and the lesion area was determined 3 dpi. The *BXL4* overexpression lines OE1, OE2, and OE3 developed smaller lesions with an average diameter of 9.5, 9.8, and 9.7 mm, respectively, compared with Col-0 with an average lesion diameter of 10.6 mm (Figure 3C). In the mock-induced *BXL4* overexpression lines, there was no significant difference in lesion size compared with Col-0 (Figure 3C). The fungal genomic DNA was quantified 3 days after spraying the plants with *B. cinerea* conidiospores. Compared with Col-0, the *BXL4* overexpression lines induced by spraying with β-estradiol once per week for 6 weeks prior to the spraying with the pathogen showed a decreased abundance of the fungal DNA (Figure 3D) and showed weaker symptoms (Figure 3E). In contrast, the transgenic plants exposed to mock induction, did not result in any significant decrease from Col-0 in fungal genomic DNA accumulation (Figure 3D) or symptoms (Figure 3E).

### Disruption of *BXL4* alters the induction of plant defense associated genes upon mechanical wounding and *B. cinerea* infection

**Figure 4:**
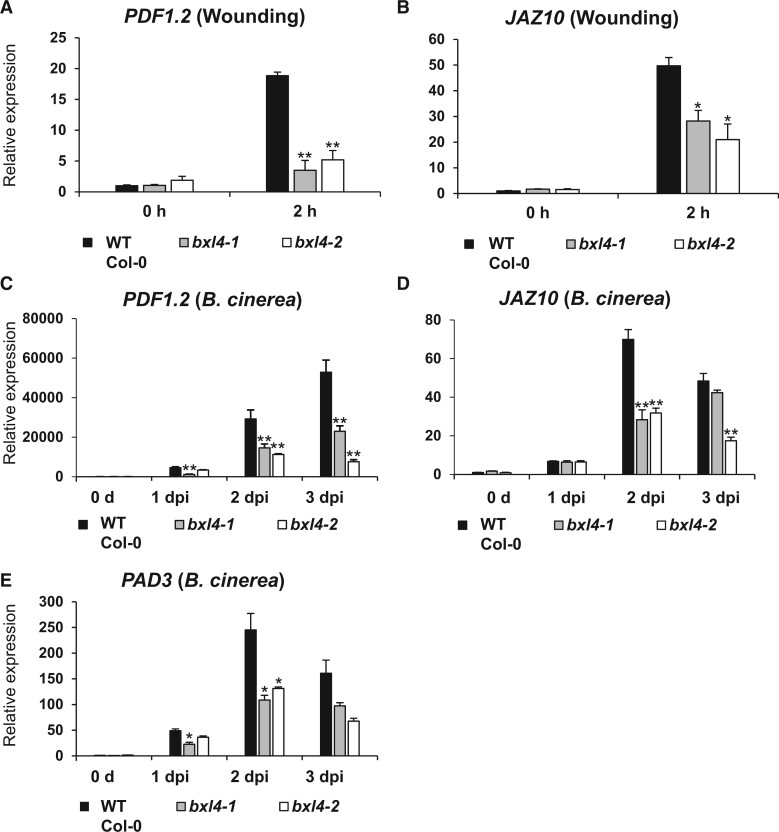
*BXL4* influences JA-Ile mediated responses upon wounding and *B. cinerea* infection. Relative expression of JA-Ile marker genes *PDF1.2* (A) and *JAZ10* (B) in Col-0, *bxl4-1*, and *bxl4-2* at 0 and 2 h post wounding. Relative expression of *PDF1.2* (C), *JAZ10* (D), and *PAD3* (E) in 6-week-old Col-0, *bxl4-1*, and *bxl4-2* Arabidopsis plants at 0, 1, 2, and 3 dpi with *B. cinerea.* Expression values were normalized to the reference gene *ACTIN8* and are shown relative to the WT levels at 0 h. Error bars show se (*n* = 3 biological replicates each performed on extracts from whole individual plants), statistical differences to the WT were determined for each time point (Student’s *t* test * indicates *P* < 0.05, **P < 0.01). Experiments were conducted three times with similar results.

Mechanical wounding and attack of necrotrophic pathogens in Arabidopsis triggers defense responses, some of which are regulated via JA-Ile signaling (Howe et al., 2018). To test if the *bxl4* mutants are impaired in JA-Ile mediated response, the transcript abundance of the JA-Ile marker genes *JASMONATE-ZIM-DOMAIN PROTEIN10* (*JAZ10*) (Yan et al., 2007; Chung et al., 2008) and *PDF1.2* (Penninckx et al., 1998; Zarei et al., 2011) was tested in mechanically wounded Col-0 and *bxl4* mutants (Figure 4, A and B) and were found to be induced to a smaller degree in the *bxl4* mutants at 2 h post wounding. The transcript accumulation of *JAZ10* and *PDF1.2* after *B. cinerea* spray inoculation was compromised in the *bxl4* mutants as well (Figure 4, C and D). This trend was also observed in three independent experiments (Supplemental Figure S5). The relative expression of the *B. cinerea*-responsive marker gene *PAD3*, encoding an enzyme involved in antimicrobial camalexin biosynthesis, was also reduced significantly in *bxl4* plants especially at 2 dpi compared with Col-0 (Figure 4E).

### *bxl4* mutant plants show a reduced accumulation of JA-Ile and camalexin after infection with *B. cinerea*

**Figure 5:**
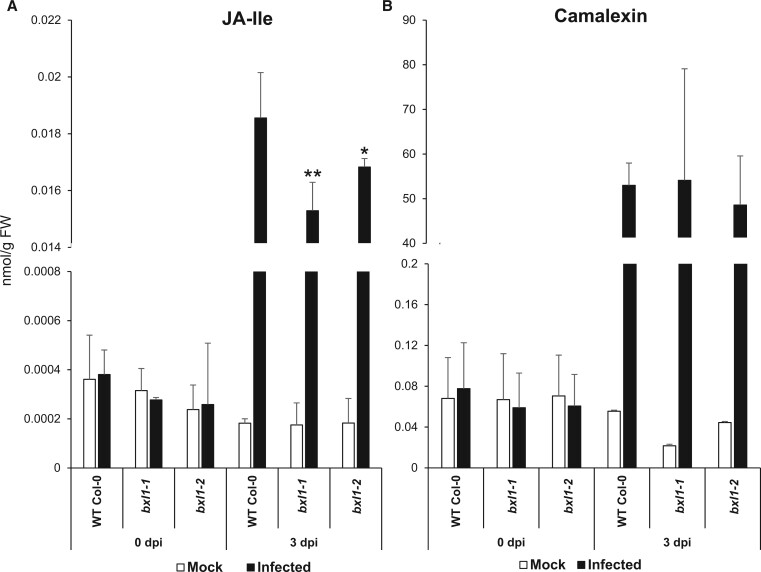
bxl4 mutants show a slightly decreased accumulation of JA-Ile and camalexin upon infection with *B. cinerea.* Col-0 and *bxl4* mutant lines were spray inoculated with *B. cinerea* and the leaves were sampled at 0, 1, 2, and 3 dpi. Extracted levels of JA-Ile (A–D) and camalexin (E–H) were analyzed using nanoelectrospray coupled to a tandem mass spectrometer. Error bars represent standard deviation of six biological replicates each performed on extracts from whole individual plants, Statistical differences to the WT were determined for each treatment (mock or infected) within each time point (Student’s *t* test * indicates *P* < 0.05, **P < 0.01). Experiments were performed three times with similar results. FW, fresh weight.

We next assayed if the reduced expression of *PDF1.2* and *PAD3* in *bxl4* mutants corresponds with a reduced accumulation of JA-Ile and camalexin upon infection with *B. cinerea* (Figure 5; Ferrari et al., 2003, 2007; Scalschi et al., 2015; Nie et al., 2017). The *bxl4* mutants showed a slight reduction in JA-Ile abundance particularly at 3 dpi compared with Col-0 (Figure 5D). The abundance of camalexin after *B. cinerea* infection was reduced especially at 1 dpi in the *bxl4* mutants compared with Col-0 (Figure 5F). There was no significant increase in the abundance of JA-Ile and camalexin after mock inoculation of the different genotypes. The abundance of other plant hormones or defense-related compounds did not show obvious alterations in *bxl4* plants after inoculation (Supplemental Dataset S1). The *bxl4* mutants also had a slightly reduced accumulation of JA-Ile compared with Col-0 after wounding (Supplemental Figure S6). Overall, however, the effects on hormones are rather subtle.

### *BXL4* overexpression results in higher expression of *PDF1.2* and *PAD3* after *B. cinerea* infection

**Figure 6:**
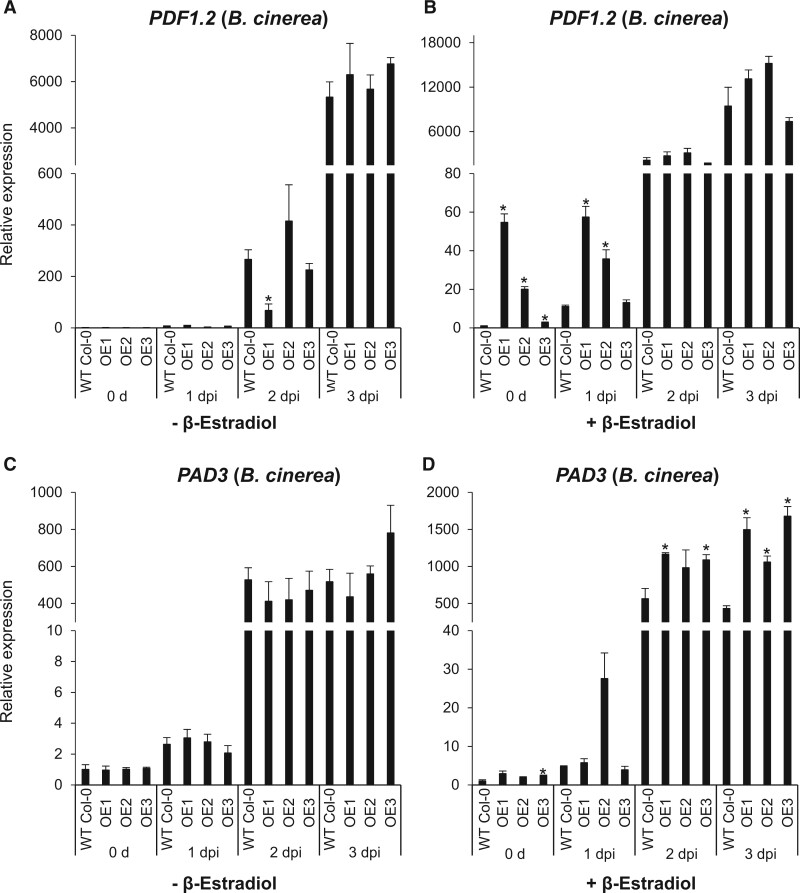
*BXL4* inducible overexpression lines display induction of *PDF1.2* and *PAD3*. Relative expression of *PDF1.2* after mock induction of *BXL4* (A) and β-estradiol induction of *BXL4* (B) in WT Col-0 and β-estradiol inducible *BXL4* overexpression lines 1, 2, and 3 (OE1, OE2, and OE3). *PAD3* expression measured in mock (C) and *BXL4* induced lines (D). Plants were induced with β-estradiol 4 days prior to *B. cinerea* inoculation and expression was measured in leaf samples collected right before (0 days) or 1, 2, and 3 dpi. Relative expression was measured by RT-qPCR, normalized to the reference gene *ACTIN8*, and relative to WT 0 h. Error bars show se of three biological replicates each performed on RNA extracted from whole individual plants; statistical differences to the WT were determined for each time point (Student’s *t* test, * indicates *P* < 0.05, **P < 0.01).

To test the effect of *BXL4* overexpression on the induction of *PDF1.2* and *PAD3* expression, the inducible *BXL4* overexpression lines were sprayed with β-estradiol or mock-induced by spraying 0.01% Tween 20, and 4 days later the plants were infected with *B. cinerea.* Mock treatment did not result in any induction of *PDF1.2* or *PAD3* prior to infection (Figure 6, A and C, time-point 0 h). Induction of *BXL4* by β-estradiol treatment, however, resulted in a strong upregulation of *PDF1.2* expression in the inducible overexpression lines compared with Col-0 (Figure 6B, time point 0 h) prior to infection. The transcript accumulation of *PDF1.2* and *PAD3* was then evaluated at 1, 2, and 3 dpi. The mock-treated overexpression lines did not show strong differences from the WT (Figure 6, A and C). When treated with β-estradiol, however, the overexpression lines had a significantly increased *PDF1.2* transcript abundance at 1 dpi (Figure 6B) and *PAD3* transcript was significantly higher, especially at 2 and 3 dpi (Figure 6D). We also tested if the overexpression of BXL4 leads to an increase in JA-Ile levels. However, JA-Ile levels were only slightly increased in one line (Supplemental Figure S7). Also the composition of the cell wall was not significantly altered upon induced overexpression of BXL4, probably because the induced changes are too subtle to measure (Supplemental Figure S8). We, therefore, tried to find evidence in a system, where BXL4 can be expressed throughout cell wall formation, that is, the seed coat epidermal cells that produce the seed mucilage during seed maturation. Seed mucilage from Arabidopsis consists predominantly of RG-I and is thought to play a role in the protection of the seed from biotic and abiotic stress as well as aiding in dispersal (Zhao et al., 2017).

### *BXL4* is able to rescue the *bxl-1* mutant phenotype in seed coat epidermal cells

**Figure 7:**
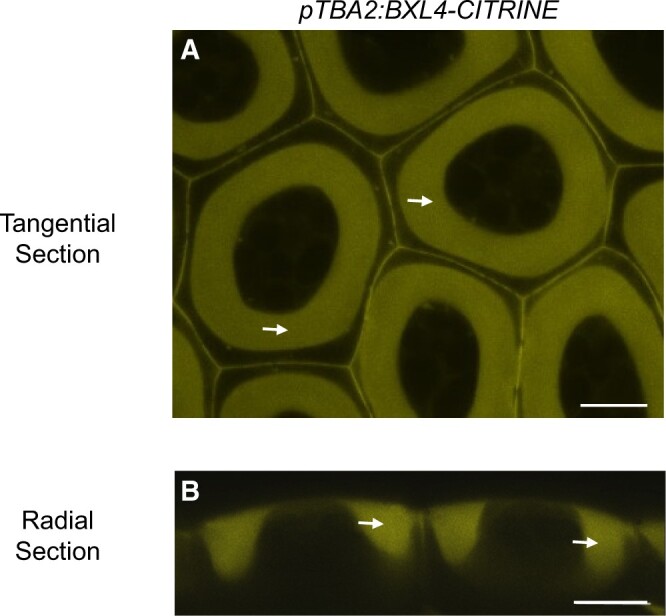
BXL4-CITRINE localizes to the apoplast in Arabidopsis Col-0 seed coat epidermal cells. BXL4-CITRINE stably expressed under the control of the TBA2 promoter localizes to the mucilage pocket (see arrows), a section of the apoplast of Arabidopsis seed coat epidermal cells, at 7 days post anthesis. Images are representative for 30 images from 18 seed coats derived from four individual lines. A, Tangential confocal section. B, radial confocal section. Scale bars, 10 µm.

BXL1, a protein closely related to BXL4, has been shown to be a β-d-xylosidase/α-l-arabinofuranosidase that removes arabinan side-chains from RG-I in Arabidopsis seed mucilage (Goujon et al., 2003; Minic et al., 2004; Arsovski et al., 2009; Williams et al., 2020). Mutations in *BXL1* result in seed mucilage with higher levels of arabinan that, following hydration, extrudes from the seed coat much more poorly than that of WT (Arsovski et al., 2009). Our analysis of the *bxl4* mutant (Supplemental Figure S3) suggests that BXL4 has a similar role in leaf pectin, but the *bxl4* mutant does not have a seed coat phenotype (Supplemental Figure S9), probably because BXL4 is not expressed there (Tsai et al., 2017). To test this hypothesis further, we sought to determine whether *BXL4* has a similar activity by testing whether it is able to rescue the *bxl1* mutant phenotype (*bxl1* is a mutant of the ecotype Wassilewskija, Ws) when ectopically expressed in seed coat epidermal cells. *BXL4* and, as a positive control, *BXL1* constructs with or without a C-terminal CITRINE fusion (*BXL4*-*CITRINE*) and under the control of the strong seed coat specific *TESTA ABUNDANT2* (*TBA2*) promoter (Tsai et al., 2017; McGee et al., 2019) were generated and transformed into Arabidopsis WT (Col-0; *pTBA2:BXL4*-*CITRINE* only) and *bxl1* mutant plants (all constructs). The seed coat epidermal cells of T2 transgenic seeds expressing *pTBA2:BXL4*-*CITRINE* or *pTBA2*:*BXL1-CITRINE* were visualized under a confocal microscope 7 days postanthesis. The BXL4-CITRINE (Figure 7 and Supplemental Figure S10) and BXL1-CITRINE (Supplemental Figure S10) fluorescence could be detected in the mucilage pocket and radial cell walls but not the cytoplasm demonstrating that our chimeric BXL4-CITRINE and BXL1-CITRINE proteins, like the endogenous BXL1, are expressed and targeted to the apoplast of seed coat epidermal cells. Other citrine fused proteins localizing to the apoplast also exhibited a similar uniform distribution in the apoplast including BETA-GALACTOSIDASE6 (BGAL6), BGAL11, BGAL16, and BGAL17 (McGee et al., 2019) and TBA1, TBA2, and TBA3 (Tsai et al., 2017).

**Figure 8:**
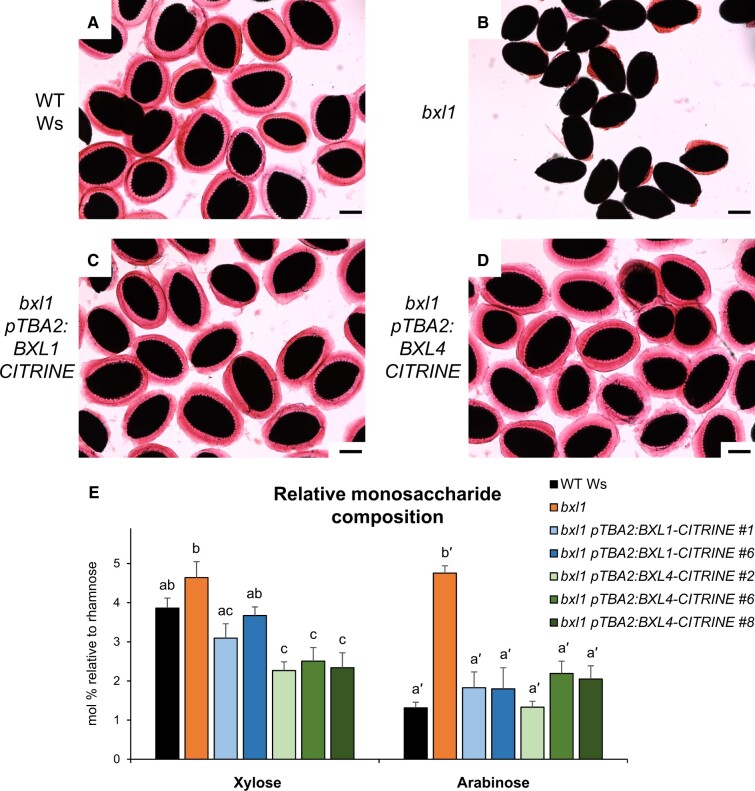
*BXL4* can complement the mucilage phenotype of *bxl1*. In Ws WT plants, the extrusion of the mucilage forms a halo around the seeds that can be visualized by staining with ruthenium red (A). *bxl1* seeds extrude their mucilage in a patchy manner (B). The *bxl1* mucilage extrusion defect can be complemented by transgenic expression of *pTBA2*:*BXL1-CITRINE* (C) or *pTBA2*:*BXL4-CITRINE* (D). Scale bars, 200 µm. Images are representative for least five images per line and for nine complemented lines. E, Relative monosaccharide composition of mucilage extracted from Ws, *bxl1, bxl1 pTBA2*:*BXL1-CITRINE* (lines 1 and 6) and *bxl1 pTBA2*:*BXL4-CITRINE* (lines 2, 6, and 8)*.* Monosaccharide composition was determined by GC–MS and normalized to rhamnose. *n* = 3 biological replicates. Error bars show sd; statistical analysis was done by one-way analysis of variance (ANOVA) with Tukey’s post hoc test. Xylose and arabinose were analyzed independently of each other; different letters indicate significant differences with *P *< 0.05.

The ability of all constructs to complement the *bxl1* mucilage extrusion defect was determined by placing T2 transgenic seeds in water and staining the mucilage with ruthenium red, a dye that stains acidic polysaccharides such as pectin (Figure 8; Steeling, 1970). Sixteen out of 17 independent transformants carrying *pTBA2*:*BXL1* (Figure 8C andSupplemental Figure S11A) and all 21 independent transformants carrying the *pTBA2*:*BXL4* (Figure 8D andSupplemental Figure S11B) constructs with and without the CITRINE tag, showed normal mucilage extrusion. In contrast, BXL6, a BXL4 homolog (Supplemental Figure S1) that is predicted to target the plasma membrane (Goujon et al., 2003) under control of the *TBA2* promoter failed to rescue the mucilage defect of *bxl1* (Supplemental Figure S12).

The Arabidopsis *bxl1* knockout mutant produces mucilage with a higher content of arabinose than the WT (Arsovski et al., 2009). To investigate if the *bxl1* transgenic lines carrying *pTBA2*:*BXL4-CITRINE* had mucilage with a monosaccharide composition more similar to the WT (Ws) than to *bxl1*, we analyzed the water-extracted mucilage from T2 seeds after mild shaking. Mucilage of Arabidopsis seeds is composed mainly of the pectin RG-I, which has a backbone alternating in galacturonic acid and rhamnose (reviewed in Šola et al., 2019a). Consistent with this, our monosaccharide analysis of the mucilage showed that rhamnose and galacturonic acid were the most abundant sugars (Supplemental Figure S13). In order to analyze changes in the side chains of RG-I, we analyzed the mucilage monosaccharide composition and normalized the values to rhamnose, one of the sugars in the RG-I backbone. The *bxl1* mutant exhibited a four-fold increase in abundance of arabinans in comparison to the WT Ws, whereas the *bxl1* transgenic lines complemented with *pTBA2*:*BXL1-CITRINE* or *pTBA2*:*BXL4-CITRINE* showed arabinose levels similar to the WT (Figure 8E). Interestingly, the *bxl1* mutant line expressing *pTBA2*:*BXL4-CITRINE* showed a xylose content that was not only reverted to the WT levels but that was even lower (Figure 8E). Taken together the data show that *BXL4* is able to completely complement *bxl1* suggesting that it has xylosidase/arabinosidase activity.

**Figure 9:**
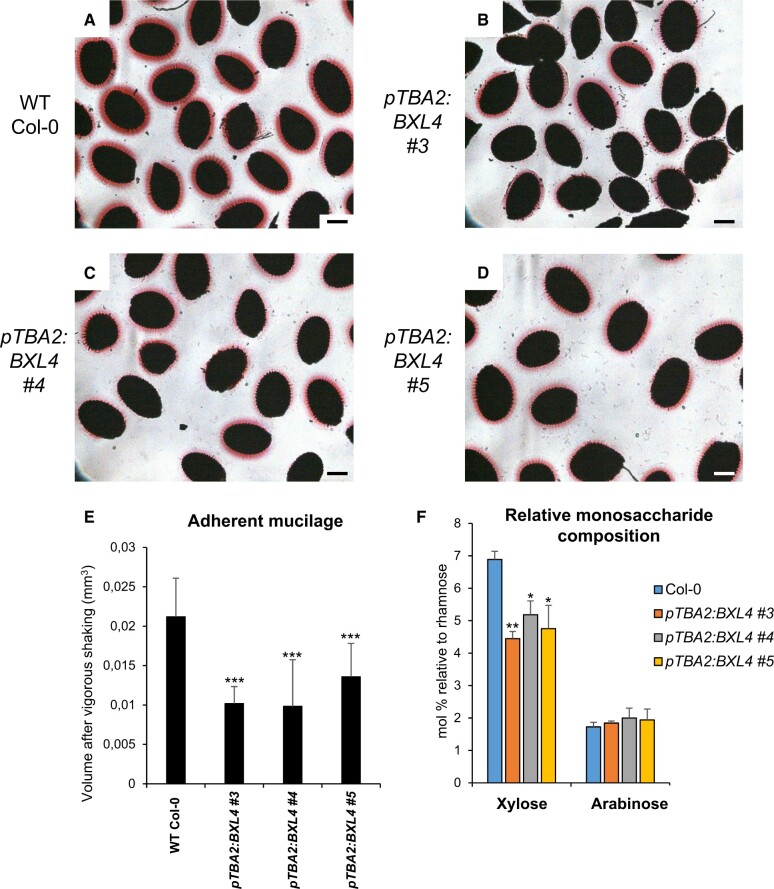
*BXL4* expression in Col-0 seed coat epidermal cells results in less adherent mucilage after vigorous shaking in water. Adherent mucilage of Col-0 (A), *pTBA2*:*BXL4* in Col-0 lines 3, 4, and 5 (B–D) after vigorous shaking in water and staining with ruthenium red. Images are representative for at least 10 images per line. Scale bar = 250 μm. E, quantification of the volume of adherent mucilage of Col-0, *pTBA2*:*BXL4* expression lines 3, 4, and 5 after vigorous shaking and staining with ruthenium red. *n *= 10 seeds. F, Monosaccharide composition of mucilage extracted from WT Col-0 and the three *pTBA2*:*BXL4* transgenic lines (3, 4, and 5). Monosaccharide composition was determined by GC–MS and normalized to rhamnose. *n* = 3 biological replicates. Error bars show sd, statistical differences to the WT were determined (Student’s *t* test; * indicates *P* < 0.05, **P < 0.01, ***P < 0.001).

As an extension to our complementation analyses, we also determined whether expression of *pTBA2*:*BXL4* in WT seeds altered the mucilage phenotype. The T2 seeds of *pTBA2*:*BXL4* transgenic plants were shaken vigorously in water for 2 h before staining with ruthenium red. The transgenic lines had an obvious reduction in the volume of adherent mucilage compared with the WT control (Figure 9, A–D) that was shown to be significant through quantification of the adherent mucilage volume using ImageJ version 1.84 (Schneider et al., 2012; Figure 9E). The mucilage from the T2 transgenic seeds was extracted and analyzed for monosaccharide composition using GC–MS. Col-0 lines expressing *pTBA2:BXL4* had lower levels of xylose compared with WT Col-0 (Figure 9F), a phenotype also observed in *bxl1-1* transformed with TBA2p:BXL4-Citrine (Figure 8E), pointing toward BXL4 having xylosidase activity. On the other hand, mucilage arabinose levels remained similar to the WT lines ectopically expressing BXL4 (Figure 9F), even though our previous data pointed toward BXL4 having arabinosidase activity (Figure 8E). Arabinose levels were similar to the WT, whilst the xylose composition was further depleted compared with Col-0 (Figure 9F).

## Discussion

The role of enzymatic pectin modifications in plant defenses has been elucidated in previous publications (Bethke et al., 2014; Lionetti et al., 2014, 2017) that highlighted for example the importance of HG methylation. Here, we provide evidence that BXL4 acts in the apoplast, can remove arabinoses and possibly xyloses from polysaccharides, and contributes to immunity against *B. cinerea.*

Our work not only investigated the role of BXL4 in pathogen defense, but also its molecular function in cell wall remodeling. The complexity of cell wall matrix polysaccharides is generated by a plethora of biosynthetic enzymes localized in the Golgi (Harholt et al., 2010). However, matrix polysaccharides can also be remodeled in muro (Micheli, 2001; Bosch, 2005) giving the plant more flexibility in the regulation of its cell wall architecture (Rui et al., 2018; Wu et al., 2018). Previous data on the cell wall proteome of Arabidopsis leaves expressing a *P. syringae* effector *AvrRpm1* indicated that BXL4 could be such an enzyme acting in muro (Breitenbach et al., 2014). The apoplast localization of BXL4 was predicted based on the algorithm of SignalP version 5.0 (Armenteros et al., 2019), which indicated that the first 38 amino acids of BXL4 act as a signal peptide for the secretory pathway. Our analyses confirm an apoplastic localization when BXL4 is introduced into seed coat epidermal cells (Figure 7 andSupplemental Figure S10) or via transient expression in *N. benthamiana* leaves (Figure 1) suggesting that BXL4 indeed has a signal sequence for secretion in vivo.

Seed coat epidermal cells were also used to investigate the enzymatic function of BXL4 (Figure 8; Šola et al., 2019a, 2019b). *BXL4*, expressed in the seed coat epidermal cells, was able to complement the mucilage extrusion and mucilage composition phenotypes of the *bxl1* mutant (Figure 8, D and E) strongly suggesting that like BXL1 (Minic et al., 2004) BXL4 has α-l-arabinofuranosidase activity and acts on the side chains present in RG-I (Williams et al., 2020). Similarly, ectopic expression of BXL4 under the strong *TBA2* promoter also significantly decreased the amount of xylose in the pectinacious mucilage especially in the Col-0 background (Figure 9F). Further, the transgenic seed also had a reduction in adherent mucilage (Figure 9, A–E), implying that the mucilage is more loosely attached to the seed coat than in the WT. These data are consistent with studies of the *MUCILAGE-MODIFIED5* (*MUM5*) gene, which encodes a xylosyl transferase. The *mum5* mutant has seed mucilage with reduced xylose levels that is more loosely attached to the seed coat (Ralet et al., 2016). It was hypothesized that mucilage RG-I has xylan side chains that mediate the interaction with mucilage cellulose through noncovalent linkages thus resulting in strong mucilage adherence to the seed surface following mucilage extrusion. It is therefore conceivable that BXL4, like BXL1 (Minic et al., 2004) also has β-d-xylosidase activity that acts on the xylan side chains of mucilage RG-I. However, we cannot rule out that other polysaccharides or glycosylated proteins are substrates of BXL4.

The expression of *BXL4* in leaves is upregulated after wounding and after infection with *B. cinerea* (Figure 1). The upregulation after wounding is partially JA-Ile dependent (Figure 1A) similar to many genes involved in wound responses and pathogen defense (Howe et al., 2018). The induction of *BXL4* expression indicates that it plays a role in cell wall remodeling after wounding and pathogen attack. Cell wall remodeling has been previously described to occur after both these stresses. For example, the degree of pectin methylesterification is altered in Arabidopsis in response to attack from fungal pathogens (Lionetti et al., 2012). In many interactions between plants and pathogens, it was noted that a high degree of methylesterification results in reduced susceptibility of the plants to pathogen as the modified pectin is more resistant to pectic enzymes of pathogens (Lionetti et al., 2012, 2017; Liu et al., 2018). Wounding is also thought to trigger the induction of endogenous polygalacturonases that generate OGs important for defense responses (León et al., 2001). Similarly, we show genetic evidence for the involvement of *BXL4* in plant biotic stress resistance, as the resistance to *B. cinerea* infection was compromised in the *bxl4* mutants (Figure 2), while the conditional overexpression of *BXL4* resulted in enhanced resistance to this pathogen (Figure 3).

While the mode of action of BXL4 in plant defense remains to be determined, upregulation of *BXL4* expression appears to be JA-Ile dependent, as detected in the *dde2-2* JA-Ile deficient mutant (Figure 1). Conversely, the loss and gain of BXL4 function influence JA-Ile-related processes since the expression levels of the jasmonate regulated genes *PDF1.2* and *JAZ10* are influenced by both overexpression and downregulation of *BXL4* (Figures 4 and 6). These data suggest that *BXL4* expression is activated by JA-Ile and in turn, BXL4 action contributes to the synthesis of JA-Ile as part of a feed-forward loop (Wasternack and Feussner, 2018). However, the changes in JA-ILE levels are only subtle and it is unlikely that BXL4 strongly influences resistance through JA-Ile related processes. More likely is that the cell wall modifications of BXL4 have a more direct effect on the ability of the pathogen to overcome the physical cell wall barrier and infect the plant tissue.

Arabinan side chains that are likely trimmed by BXL4 play a role in cell wall architecture (Verhertbruggen et al., 2013). In Arabidopsis, the interspacing of HG with arabinan-rich RG-I reduces crosslinking with Ca^2+^, thus making cell walls more flexible (Jones et al., 2003; Moore et al., 2008; Merced and Renzaglia, 2019). Trimming of arabinan side chains in WT plants could result in greater pectin crosslinking by Ca^2+^ thus making the cell walls more recalcitrant to penetration by fungal hyphae. In addition, the degradation of this cross-linked homogalacturan by polygalacturonases (Bellincampi et al., 2014; Sénéchal et al., 2014) would result in the formation of Ca^2+^ cross-linked OGs, which elicit strong biological responses such as the production of reactive oxygen species, phytoalexins, callose, and JA-Ile production (Kohorn and Kohorn, 2012; Bethke et al., 2014; Savatin et al., 2014; Mielke and Gasperini, 2019). It is also conceivable that changes in the cell wall composition induced by *BXL4* are perceived by plasma membrane-bound receptor kinases resulting in the activation of plant defense responses (Bacete et al., 2018; Engelsdorf et al., 2018; Franck et al., 2018). Furthermore, BXL4 activity on the cell wall polysaccharides may result in the generation of other nonpectic fragments, for instance, xyloglucan derived fragments and cellulose derived oligosaccharides which act as danger signals that trigger plant immune responses (Claverie et al., 2018; Zarattini et al., 2021). The mutants with a compromised BXL4 activity may therefore, be unable to produce these danger signals resulting in an impaired immune response, thus leading to increased susceptibility to *B. cinerea*. One of the mutants, *bxl1-2* exhibits a reduction in fucose in its leaf AIR composition (Supplemental Figure S3). This could possibly explain the increased susceptibility to *B. cinerea* as Molina et al. (2021) could demonstrate that Arabidopsis mutants with increased abundances of fucosylated xyloglucans were more resistant to the necrotrophic fungus *Plectosphaerella cucumerina*. The Arabidopsis mutant *murus1* (*mur1*) with no cell wall fucose also has increased susceptibility to *P.* *syringae* DC3118 (Zhang et al., 2019). Modifications in other cell wall components not identified in this research and their degradation fragments could have also contributed to the disease phenotype observed in this research. It has been demonstrated that the alteration of cell xylose content alters resistance to different pathogens. Arabidopsis mutants with increased xylose content for example *de-etiolated3* (Rogers et al., 2005) show a significantly higher resistance to *P.* *cucumerina*, while mutants with reduced cell wall xylose content have increased susceptibility to the same plant pathogen (Delgado-Cerezo et al.,2012).

Another gene influenced by BXL4 and partially regulated by JA-Ile signaling is *PAD3* (Rowe et al., 2010) that catalyzes the final step in camalexin biosynthesis (Schuhegger et al., 2006). Camalexin has been shown to act as a phytoalexin not only against *B. cinerea* (Ferrari et al., 2007; Shlezinger et al., 2011) but also various other phytopathogens (Sanchez-Vallet et al., 2010; Schlaeppi et al., 2010). The reduction in the accumulation of camalexin in *bxl4* mutants, especially at early time points during infection (Figure 5F), might be one small additional factor that leads to enhanced susceptibility to *B. cinerea*, even though the differences are rather small and can only be found in early time-points.

## Conclusion

Findings from our study indicate that the modification of cell wall polysaccharides by BXL4 is a factor that influences plant defense responses to the necrotrophic fungus *B. cinerea*.

## Materials and methods

### Plant and *B. cinerea* growth conditions

Arabidopsis (*A.* *thaliana*) plants used for infection assays were grown on semi-sterile soil heated in an oven at 80°C for 8 h. The plants were grown under short-day conditions (8 h light and 16 h darkness) at a temperature of 22°C and a relative humidity of 65% in a growth cabinet (Percival Scientific, Perry, GA, USA). Arabidopsis plants for seed propagation were grown under long-day conditions (16 h light and 8 h darkness), light intensity of 120–150 μmol m^−2^ s^−1^, at 22°C and 60% relative humidity in a climate chamber (York Industriekälte, Mannheim, Germany). The *bxl1-1* mutant and lines created from this mutant were in the Ws ecotype background. All other lines used in this study were in the Col-0 ecotype background. *bxl1-1* (Ws ecotype; CS16299, Feldmann, 1991), *mpk3*-DG (Li et al., 2002), and *dde2-2* (von Malek et al., 2002) were used. T-DNA mutant lines of *bxl4-1* (SALK_071629) and *bxl4-2* (SAIL_331_B06) were sourced from Nottingham Arabidopsis Stock Centre and homozygous mutants were confirmed through genotyping PCR on genomic DNA using REDTaq ReadyMix (Sigma, St. Louis, MO, USA) following their protocol. Primers used are listed in Supplemental Table S1.

Spores of the *B. cinerea* strain B05-10 (Staats and van Kan, 2012) were cultured on potato dextrose broth (Sigma) plus agar, grown at RT for 10 days and harvested by washing the spores off the plates using 1/4 potato dextrose broth and sieving through Miracloth (Sigma) to collect the conidiospores. Conidiospores were counted using a hemacytometer (Sigma) and stocks in 25% (v/v) glycerol were made and stored at −80°C.

### Wounding assay

Rosette leaves from 6-week-old Arabidopsis plants grown under short-day conditions (8-h light and 16-h darkness) were wounded with a forceps as described in Stenzel et al. (2003). The rosettes were then harvested at different time points and immediately frozen in liquid nitrogen before RNA extraction.

### Gene expression analysis (RT-qPCR)

RNA was extracted from leaves using Spectrum Plant Total RNA Kit (Sigma). cDNA was made from RNA treated with DNaseI (Thermo Scientific, Waltham, MA, USA) using Revert Aid H minus Reverse Transcriptase (Thermo Scientific). cDNA derived from leaf RNA was used for RT-qPCR using Takyon No Rox SYBR MasterMix dTTP Blue (Eurogentec, L;ϋttich, Belgium) following the manufacturers’ instructions. The 2^−△△CT^ method (Livak and Schmittgen, 2001) was used to estimate relative gene expression which was normalized to the reference gene *ACTIN8* (Ralhan et al., 2012). The primers used are listed in Supplemental Table S1.

### Molecular cloning and Arabidopsis transformation

The R4 Gateway Binary Vectors (R4pGWB; Nakagawa et al., 2008) were employed to make several constructs used in this work. The chimeric constructs with and without a Citrine tag were assembled by first amplifying the *TBA2* promoters (1,293 bp) using PCR and cloning into the entry vector pDONRP4-P1R. The cDNA constructs were made by amplifying the cDNA from WT Arabidopsis plants with PCR (primers used are shown in Supplemental Table S1) and cloning into entry vector pDONR207 (Invitrogen, Waltham, MA, USA). A tripartite LR reaction was performed to incorporate the *TBA2* promoter and cDNA into R4pGWB501 (modified vector) with and without Citrine tag (Nakagawa et al., 2008). The *BXL4* inducible overexpression lines were generated using pER8-GW-3’HAStrep, a plant binary gateway destination/35S-inducible expression vector with a pER8-vector backbone (Breitenbach et al., 2014). Arabidopsis plants were transformed by floral dipping as described (Clough and Bent, 1998) and the subsequent T1 seeds were germinated on Murashige and Skoog medium supplemented with hygromycin for selection. The induction of *BXL4* in the inducible *BXL4* overexpression lines was carried out by spraying 6 weeks old Arabidopsis plants with 50 µM β-estradiol in 0.01% (v/v) Tween20. Mock induction was performed by spraying the lines with 0.01% Tween20.

### Confocal microscopy

The transformed Arabidopsis T2 seeds were visualized using a confocal microscope for localization of BXL proteins. Confocal images were recorded using confocal microscope Zeiss LSM 780 with a 63× objective (Carl Zeiss Inc., Jena, Germany). Citrine was excited at 488 nm through a 488 nm major beam splitter. Detection of fluorophore was done at a wavelength of 514–530 nm and a gain of 700. Micrographs of *N. benthamiana* leaves infiltrated with *Agrobacterium* *tumefaciens* for transient expression were acquired using a Zeiss LSM710 confocal laser-scanning microscope equipped with a Apochromat 40× objective lens. BXL4-CITRINE and plasma membrane marker PIP2A:mCherry (Nelson et al., 2007) were excited at a wavelength of 561 and 514 nm, respectively, while the emission fluorescence signals were collected at 537 and 632 nm, respectively, at a gain of 600 and 578, respectively. Excitations and emission signals for fluorescent proteins were collected sequentially.

### *Botrytis cinerea* infection assay

*Botrytis* *cinerea* spores were diluted to 5 × 10^4^ spores per milliliter in Vogel buffer (Vogel, 1956) for drop inoculation assay or 2 × 10^5^ spores per milliliter in Vogel buffer for spray inoculation assay used for RT-qPCR analysis. The spores were pregerminated for 4 h before inoculations were carried out. For drop inoculations, 6 µL of spore suspension in Vogel buffer was carefully placed on the adaxial side (away from the midrib) of a fully expanded rosette leaf of 6–7 week-old Arabidopsis plants (at least 30 leaves were used from 10 independent plants). For spray inoculation, plants were sprayed until droplets began to run off the leaves (Mengiste et al., 2003). Inoculated plants were covered and grown under high humidity conditions for 3 or 4 days. Lesion diameters of drop-inoculated leaves were measured using a digital caliper and used to calculate lesion diameter. Spray-inoculated rosette leaves were harvested at 3 dpi. For fungal DNA quantification, fungal DNA was extracted using a plant/fungi DNA isolation kit (Norgen Biotek Corp, Thorold, Ontario, Canada) following the manufacturer’s protocol. The fungal β*-ACTIN* genomic DNA was quantified by qPCR (Ettenauer et al., 2014) using primers listed in Supplemental Table S1.

### Mucilage staining with ruthenium red

Five micrograms of Arabidopsis seeds were placed in 500 µL _dd_H_2_O in an Eppendorf tube before being gently shaken for 1 h on a rotary shaker. Water was gently removed and 500 µL 0.02% ruthenium red (Sigma) was added (Dean et al., 2007) to the tubes, which were then shaken for another 15 min before the ruthenium red solution was removed and seeds were again resuspended in 500 µL _dd_H_2_O. A droplet with stained seeds was placed on a microscopic slide and viewed under a light microscope (BX51, Olympus, Shinjuku, Japan, equipped with an R6 Retiga camera, Q imaging, Surrey, BC, Canada) using a 4× objective.

### Monosaccharide analysis of mucilage

Mucilage was extracted by agitating 5 mg of seeds in water for 2 h on a rotary shaker. Seeds were then allowed to settle for a few minutes before 1 mL mucilage solution was removed and placed in a glass tube. Mucilage solution was evaporated in a water bath at 40°C under nitrogen stream. Dry samples were hydrolyzed for 1 h at 121°C using 2 M trifluoroacetic acid, before being evaporated again. About 100 µL *allo*-inositol was added as an internal standard and 500 µL _dd_H_2_O was added to resuspend the hydrolyzed mucilage. About 20 µL sample was evaporated under a nitrogen stream before overnight derivatization in 15 µL methoxyamide (30 mg/mL in anhydrous pyridine). The next day, 30 µL *N*-methyl-*N*-(trimethylsilyl)trifluoroacetamide (MSTFA) was added, and samples were analyzed using GC–MS 1–6 h after MSTFA addition.

### GC–MS analysis

Samples were analyzed with a 7890B GC-System coupled to a 5977B MSD quadrupole set-up from Agilent Technologies. GC-separation was achieved on a HP-5 column (Agilent Technologies, Santa Clara, CA, USA) using the following temperature gradient: 150°C for 2 min, 5 K/min gradient for 20 min, 15 K/min to a final temperature of 320°C, which was held for 3 min. For each run, 1 µL of the derivatized sample was injected. Identification of compounds was done by a combination of retention times compared with external standards and MS spectra. Prepared mucilage samples were quantified relative to the internal standard *allo*-inositol. In parallel runs, monosaccharide standards of different concentration were used to determine response factors for area-to-molar amount conversion allowing absolute quantification.

### Monosaccharide analysis of the AIR of leaves

The AIR was extracted from plant leaves (6–7 weeks old plants) grown in the dark 2 days before harvesting the plant samples to reduce starch that could interfere with the measurements. The AIR was extracted as described (Gille et al., 2009). The leaves were flash frozen in liquid nitrogen before they were pulverized using mortar and pestle. The ground material was washed two times with 70% (v/v) ethanol, washed thrice with a chloroform:ethanol mixture (1:1 [v/v]), and lastly with acetone, before being air dried. Hot water pectin extraction (Yeoh et al., 2008) was used by shaking 2 mg of AIR in 1.4 mL _dd_H_2_O at 90°C for 2 h. Monosaccharide analysis was then carried out on AIR using the same GC–MS method used on mucilage as described above.

### Calculation of adherent mucilage volume

The shape of the seed was taken as a spheroid as described in Yu et al. (2014). The total length (2A) and width (2B) of the seed including the mucilage was measured and the volume calculated. The length of the seed alone without mucilage (2a) and the width without mucilage (2b) was measured and used to calculate the volume of the seed. The volume of the adherent mucilage was calculated by subtracting the volume of the seed alone from the total volume of the seed with mucilage using the formula: volume = 4/3 × 1/8 × length × width^2^ (Supplemental Figure S14).

### Pectin dot blot assay

The dot blot assay was performed as described in Bethke et al. (2016). Pectin was extracted using a pectin extraction buffer (50 mM Trizma and 50 mM CDTA, pH 7.2) at 50 µL/mg AIR. Serial dilutions were done before spotting 1 µL on nitrocellulose membranes. Overnight drying of the membrane was done before the membranes were blocked by adding 5% milk powder (w/v) dissolved in 1× PBS (8 g L^−1^ NaCl, 0.2 g L^−1^ KCl, 1.44 g L^−1^ Na_2_HPO_4_, and 0.24 g L^−1^ KH_2_PO_4_, pH 7.4). The membranes were probed with LM13, an anti-arabinan (Verhertbruggen et al., 2009), LM19, an anti-unesterified-HG (Verhertbruggen et al., 2009), and CCRC-M7, an anti-RG-I (Steffan et al., 1995) antibodies. LM13 and LM19 antibodies were diluted 1:250 and CCRC-M7 diluted 1:500 in 5% milk powder (w/v) dissolved in 1× PBS. Rabbit anti-rat IgG antibody (Sigma) diluted 1:30,000 in 5% milk powder (w/v) in 1× PBS was used for the LM antibodies. Goat anti-mouse IgG antibody (Sigma) diluted 1:30,000 in 5% milk powder (w/v) in 1× PBS was used for CCRC-M7 antibodies. Blots were developed by equilibrating in AP buffer (100 mM Tris, 100 mM NaCl, 5 mM MgCl_2_, pH 9.5) before incubating in 10 mL AP buffer with 33 µL BCIP and 66 µL NBT in the dark until spots were visible.

### Phytohormone measurements

Extraction of phytohormones, separation, and analysis were carried out as described in Herrfurth and Feussner (2020) using the described mass transitions with some modifications specified in Supplemental Table S2.

### Creation of phylogenetic tree

Phylogenetic trees were created with MEGA version X software using MUSCLE alignment with gap penalties set to −9 for gap open and to −3 for gap extension (Kumar et al., 2018). The aligned protein sequences were used for phylogenetic tree construction using the maximum-likelihood method based on the JTT matrix-based model (Jones et al., 1992). The phylogeny was tested with the Bootstrap method set for 1,000 replicates (Felsenstein, 1985).

## Accession numbers

Sequence data from this article can be found in the GenBank/EMBL data libraries under accession numbers *BXL1* (AT5G49360), *BXL4* (AT5G64570), *BXL6* (AT5G10560), *PDF1.2* (AT5G44420), *PAD3* (AT3G26830), and *JAZ10* (AT5G13220).

## Supplemental data

The following materials are available in the online version of this article.

**Supplemental Table S1.** List of primers.

**Supplemental Table S2.** Mass transitions and corresponding conditions for determination of the phytohormones.

**Supplemental Figure S1.** Phylogenetic tree of BXLs from *A.* *thaliana*.

**Supplemental Figure S2.** Expression of *BXL4* in Arabidopsis rosette leaves.

**Supplemental Figure S3.** The disruption of BXL4 has mild effects on the leaf cell wall composition.

**Supplemental Figure S4.** Morphological phenotypes of Col-0, *bxl4-1*, and *bxl4-2*.

**Supplemental Figure S5.** BXL4 acts upstream of JA-Ile-mediated responses upon *B. cinerea* infection.

**Supplemental Figure S6.** JA-Ile accumulation after mechanical wounding of Arabidopsis leaves.

**Supplemental Figure S7.** Induction of BXL4 induces a slight accumulation of JA-Ile in Arabidopsis.

**Supplemental Figure S8.** Monosaccharide composition of pectin extracted from leaf AIR.

**Supplemental Figure S9.** *bxl4* mutants show WT-like extrusion of mucilage.

**Supplemental Figure S10.** BXL1-CITRINE and BXL4-CITRINE localize to the apoplast in Arabidopsis *bxl1* seed coat epidermal cells.

**Supplemental Figure S11.** *BXL4* without a CITRINE tag complements the mucilage phenotype of *bxl1*.

**Supplemental Figure S12.** BXL6 fails to complement the mucilage phenotype of *bxl1*.

**Supplemental Figure S13.** Mucilage monosaccharide composition.

**Supplemental Figure S14.** Calculation of adherent mucilage volume.

**Supplemental Dataset S1.** Phytohormone analysis.

## Supplementary Material

kiac165_Supplementary_Data

## Notes

A.G., A.C.V., G.H., M.Wiermer, I.F., and T.I. designed the research. A.G., R.M., D.H., P.S., C.H., K.B., M.Wenig, D.L., and K.Z. performed the experiments. M.Wiermer, A.C.V., I.F., G.H., and T.I. analyzed the data. A.G. and T.I. wrote the manuscript with contributions from the other authors.

The author responsible for distribution of materials integral to the findings presented in this article in accordance with the policy described in the Instructions for Authors (https://academic.oup.com/plphys/pages/general-instructions) is: Till Ischebeck (till.ischebeck@uni-muenster.de).
