# Supplementary material for: Cell wall-localized BETA-XYLOSIDASE4 contributes to immunity of Arabidopsis against Botrytis cinerea
Source: Plant Physiol. 2022 Apr 18;189(3):1794–813. doi: 10.1093/plphys/kiac165 (PMC9237713; doi:10.1093/plphys/kiac165)
Supplement: kiac165_Supplementary_Data [file kiac165_supplementary_data.zip › PP2021RA01704R1_Supplemental_Figures_S1_S14.pdf]

Supplementary tables and figures

Guzha et al.

# Cell wall-localised BETA-XYLOSIDASE4 contributes to immunity of Arabidopsis against *Botrytis cinerea*

**Supplemental table S1: List of primers**

| **Cloning primers** | **Forward 5'-3'** | **Reverse 5'-3'** |
|---|---|---|
| *BXL1* | GGGGACAAGTTTGTACAAAAAAGCAGGCTATGTCTTGTTATAATAAAGCACTATTG | GGGGACCACTTTGTACAAGAAAGCTGGGTAAAGTTGCGGTTGGACCAA |
| *BXL4* | GGGGACAAGTTTGTACAAAAAAGCAGGCTTGGGCTCTTCTTCTCCATTA | GGGGACCACTTTGTACAAGAAAGCTGGGTAGATTCTAATGCTTAAGGAATGTTTTA |
| *BXL6* | GGGGACAAGTTTGTACAAAAAAGCAGGCTATGAATCTTCAGTTGACTCTAATC | GGGGACCACTTTGTACAAGAAAGCTGGGTAGAATTCAACAGAGAGAGAATGT |
| *BXL4* (OE) | CACCATGGGCTCTTCTTCTCC | GATTCTAATGCTTAAGGAATGTTTTAAATCTCCG |
| **Genotyping primers** | **LB** | **RB** |
| *bxl1* | ATTTTGCCGATTTCGGAAC (LBb1.3) | AACCGTCGCGTCGGCTTCAC |
| *bxl4-1* | LBb1.3 | ATCTCCGACATGAAGAAGATGC |
| *bxl4-2* | TAGCATCTGAATTTCATAACCAATCTCGATAC | ATCTCCGACATGAAGAAGATGC |
| *bxl6* | LBb1.3 | TACCACAGCATTGAAGTCGTATC |
| **RT-qPCR primers** | **Forward 5'-3'** | **Reverse 5'-3'** |
| *BXL4* | GGTGGATCGGCGTTTGAAGT | TACCAAATGCTTCCTCGGCG |
| *BXL4a* | TCAACGCCGTGGTGACTCAA | CGCATGTCGGTTTGCCGTTA |
| *BXL4b* | CCCACACCTGTTTTCAGTGCC | TACATTGCCCTCGCTTCCGT |
| *PDF1.2* | TTGCTGCTTTCGACGCA | TGTCCCACTTGGCTTCTCG |
| *JAZ10* | ATCCCGATTTCTCCGGTCCA | ACTTTCTCCTTGCGATGGGAAGA (Benthke et al., 2016) |
| *PAD3* | TGCTCTCAAGTTCACCACT | CGAATCTCGTCTTGCACTT (Benthke et al., 2016) |
| *ACTIN8* | GGTTTTCCCCAGTGTTGTTG | CTCCATGTCATCCCAGTTGC (Ralhan et al., 2012) |
| *Botrytis ACTIN* | TGGAGATGAAGCGCAATCCA | AAGCGTAAAGGGAGAGGACG |
| *Botrytis TUBULIN* | CCGTCATGTCCGGTGTTAC | CGACCGTTACGGAAATCGG |

**Supplemental Table S2: Mass transitions and corresponding conditions for determination of the phytohormones.**

| MRM Transitions | | Analyte | DP [declustering potential] | EP [entrance potential] | CE [collision energy] |
|---|---|---|---|---|---|
| Q1 | Q3 | | | | |
| 209 | 59 | JA | -30 | -4.5 | -24 |
| 225 | 59 | 11,12-0H-JA | -35 | -9 | -28 |
| 263 | 165 | dinor-oPDA | -40 | -5 | -20 |
| 296 | 170.2 | D5-oPDA | -65 | -4 | -28 |
| 305 | 97 | 12-HS04-JA | -30 | -4 | -32 |
| 308 | 116 | JA-Val | -45 | -5 | -28 |
| 322 | 130 | JA-lle/Leu | -45 | -5 | -28 |
| 325 | 133 | D4-JA-Leu | -80 | -4 | -30 |
| 324 | 116 | 120H-JA-Val | -45 | -10 | -30 |
| 338 | 130 | 120H-JA-lle | -45 | -10 | -30 |
| 352 | 130 | 12COOH-JA-lle | -45 | -10 | -30 |
| 387 | 59 | 12-0-Gluc-JA | -85 | -9 | -59 |

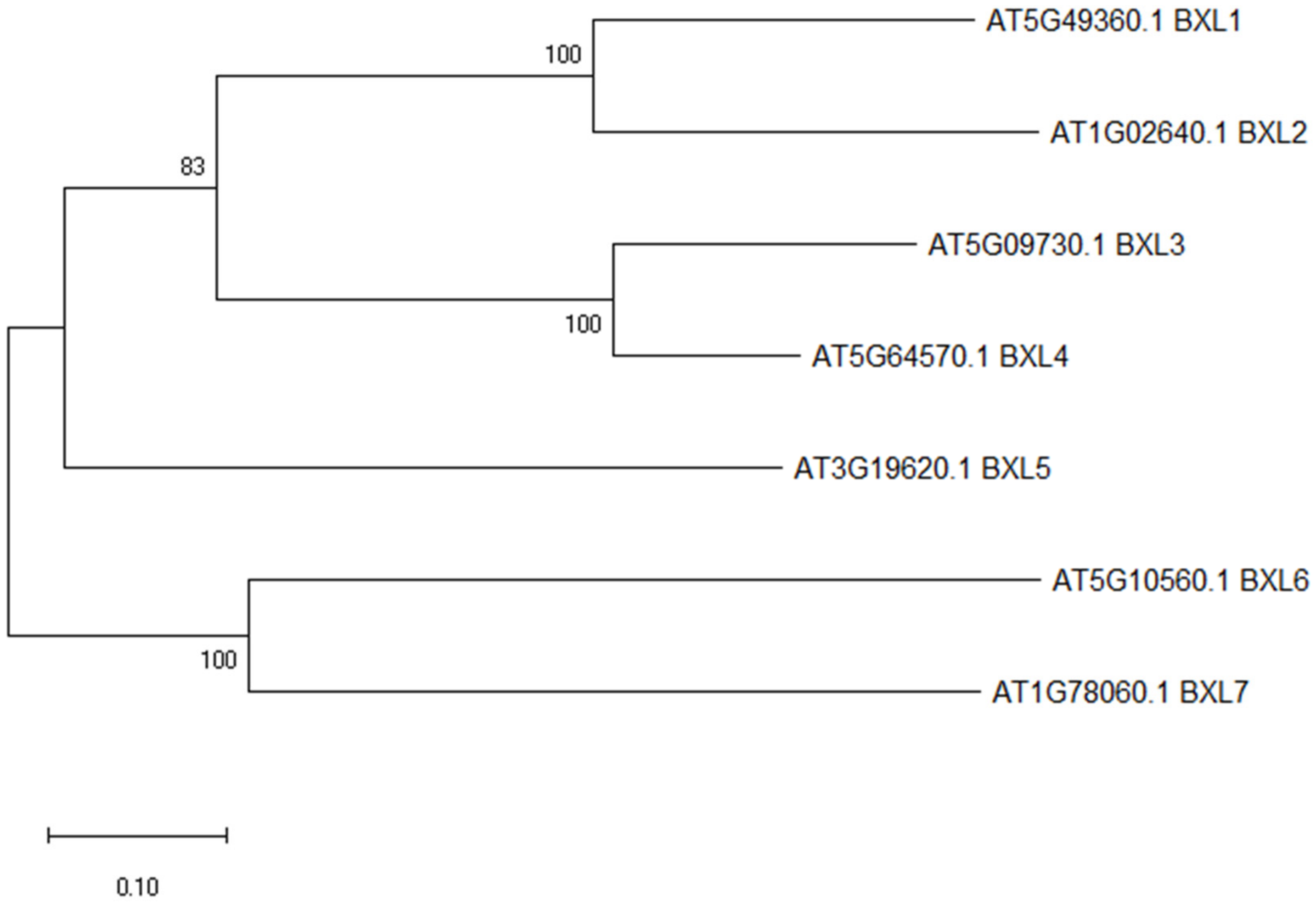


**Supplemental Figure S1. Phylogenetic tree of betaxylosidases (BXLs) from *Arabidopsis thaliana*.**
The evolutionary history was inferred by using the Maximum Likelihood method based on the JTT matrix-based model (Jones et al., 1982). The tree with the highest log likelihood (-9603.16) is shown. The percentage of trees in which the associated taxa clustered together is shown next to the branches. Initial tree(s) for the heuristic search were obtained automatically by applying Neighbor-Join and BioNJ algorithms to a matrix of pairwise distances estimated using a JTT model, and then selecting the topology with superior log likelihood value. The tree is drawn to scale, with branch lengths measured in the number of substitutions per site. The analysis involved 7 amino acid sequences. There were a total of 828 positions in the final dataset. Evolutionary analyses were conducted in MEGA X (Kumar et al., 2018). Scale bar, 0.10 number of substitutions per site.

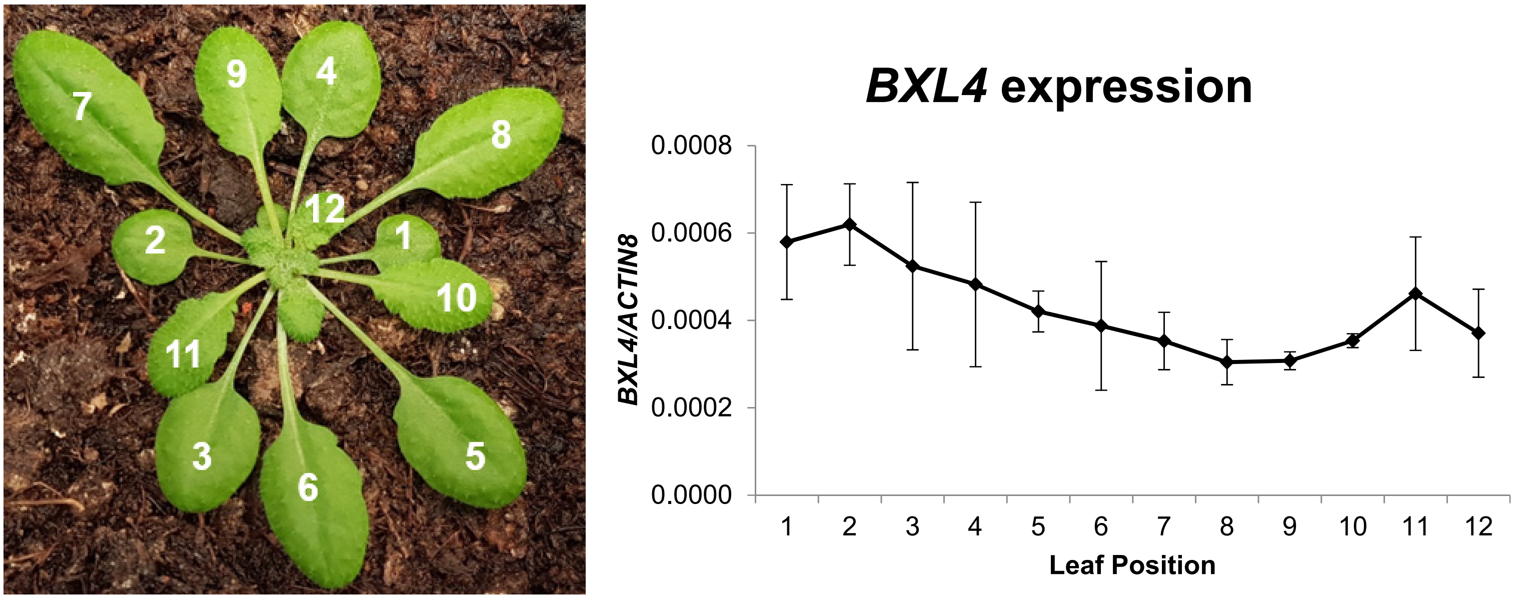


**Supplemental Figure S2. Expression of *BXL4* in Arabidopsis rosette leaves.**
RNA was extracted from the different Arabidopsis (Col-0) leaves starting from the oldest rosette leaf (1) to the youngest (12). Expression of *BXL4* was determined by RT-qPCR and normalised to the expression of *ACTIN8*. Error bars represent SE of three biological replicates each performed on RNA extracted from single leaves individual plants.

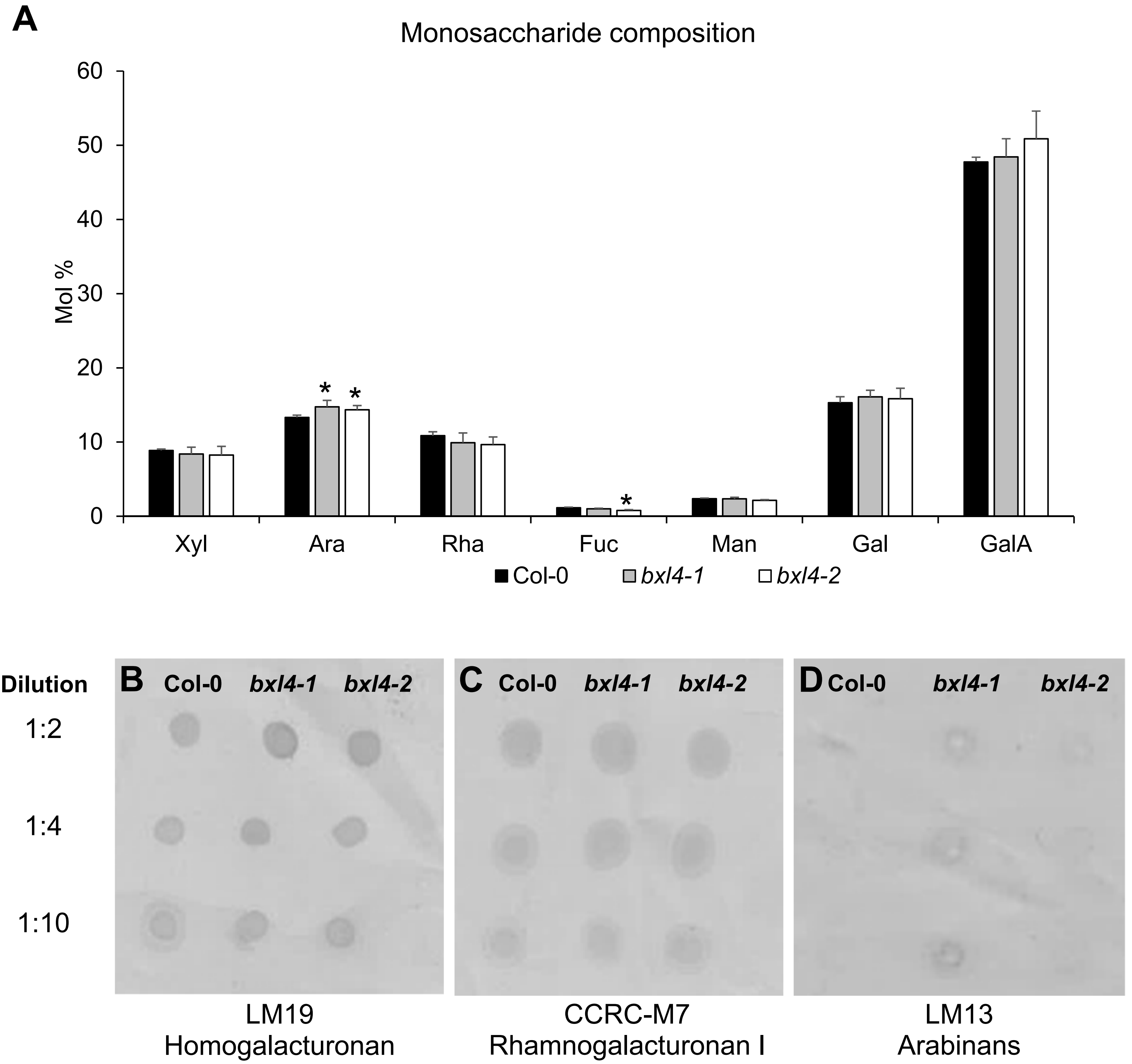


**Supplemental Figure S3. The disruption of *BXL4* has mild effects on the leaf cell wall composition.** Monosaccharide composition of water extracted pectin from Arabidopsis leaves of 6 week old wildtype Col-0, *bxl4-1* and *bxl4-2* mutant lines. The monosaccharides were normalised to total sugars. Extracted pectin was analysed by GC-MS. Error bars show SD (n=4 biological replicates), statistical differences to the WT were determined (Student's *t*-test, * indicates $P<0.05$). Experiments were conducted three times with similar results. Pectin was extracted from the AIR of the three indicated genotypes using pectin extraction buffer. The pectin was serially diluted before 1 µL was spotted onto a nitrocellulose membrane. The dot blot was carried out using the antibodies LM19 (B), CCRC-M7 (C) and LM13 (D) that target the cell wall components as indicated.

3 weeks

**A**

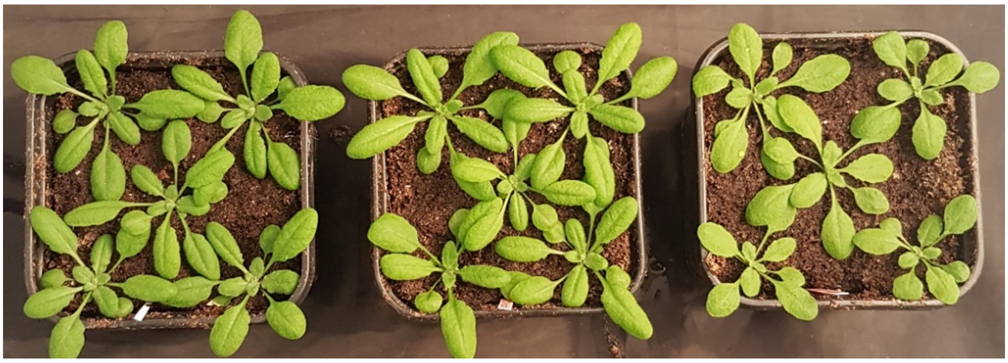

8 weeks

**B**

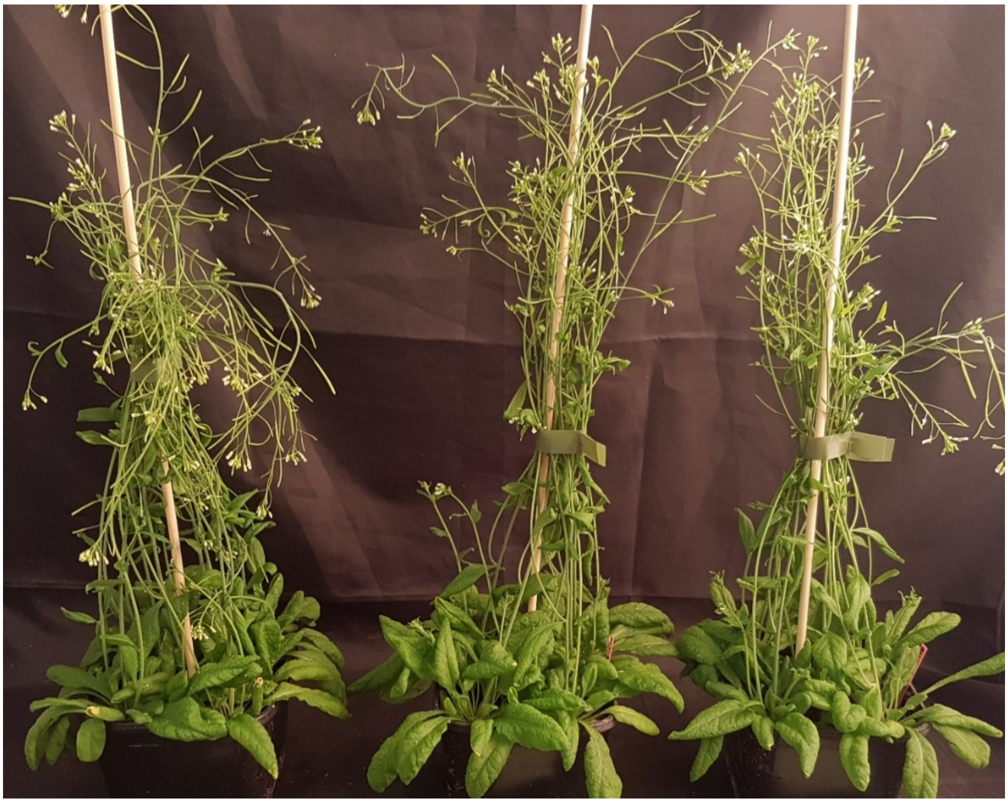

**WT Col-0** ***bxl4-1*** ***bxl4-2***

**Supplemental Figure S4. Morphological phenotypes of Col-0, *bxl4-1* and *bxl4-2*.**
The morphological phenotypes of 3 weeks (A) and 8 weeks old (B) Col-0, *bxl4-1* and *bxl4-2* grown under long day conditions (16 h light/8 h dark).

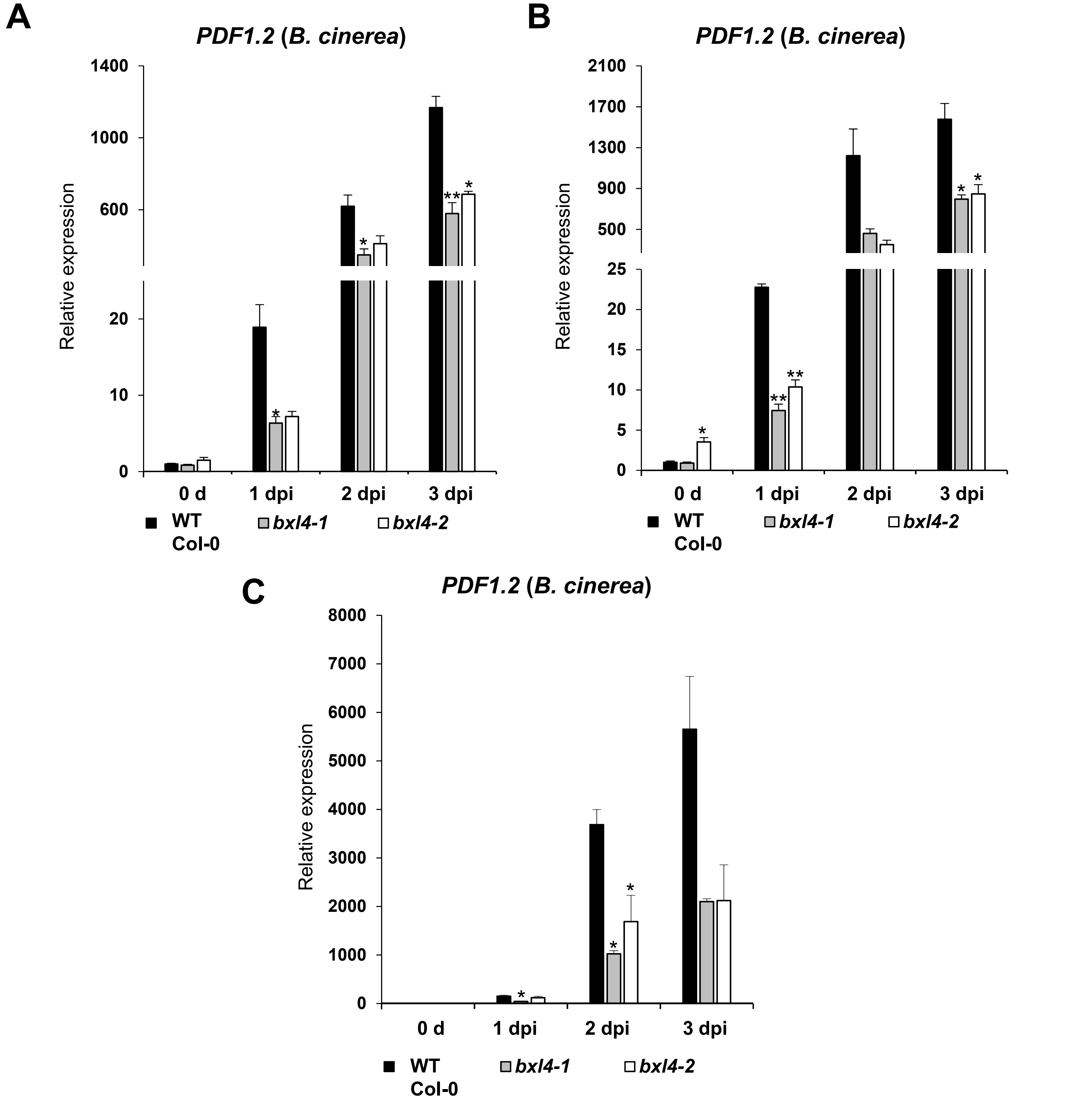


**Supplemental Figure S5. BXL4 acts upstream of JA-Ile mediated responses upon *B. cinerea* infection.**

Relative expression of *PDF1.2* in RNA extracted from 6 week old WT Col-0, *bxl4-1* and *bxl4-2* Arabidopsis plants at right before (0 d) or 1, 2 and 3 days post infection (dpi) with *B. cinerea.* Data shown is from 3 independent experiments (A-C). Expression values were normalised to the reference gene *ACTIN8* and are shown relative to the wild type 0 h levels. Error bars show SE (n=3 biological replicates each performed on RNA extracted from whole individual plants), statistical differences to the WT were determined for individual time points (Student's *t*-test, * indicates P<0.05, ** P<0.01).

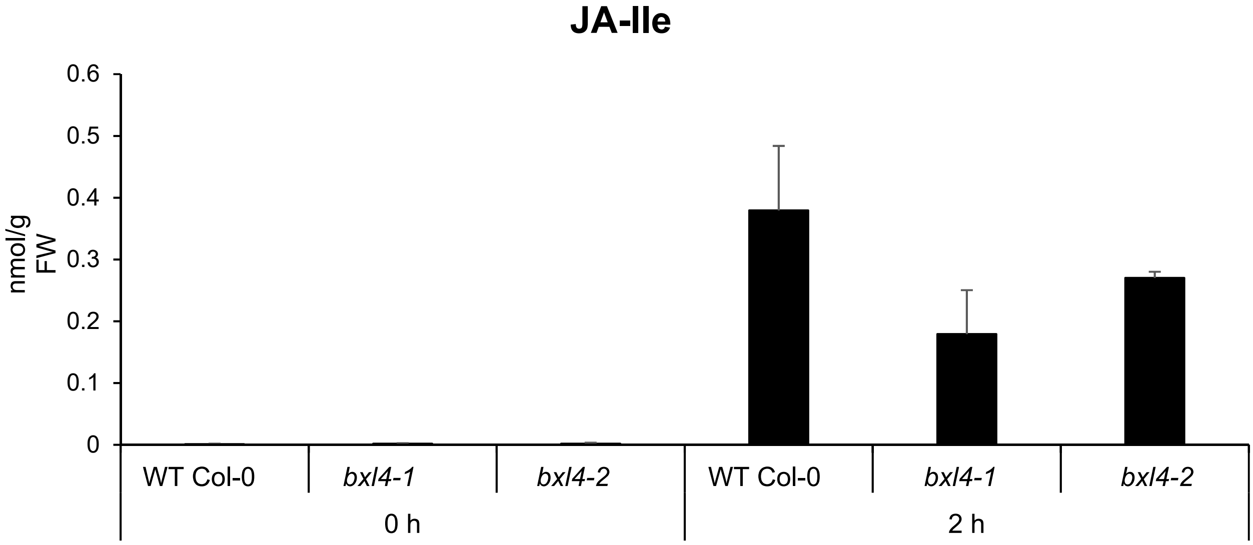


**Supplemental Figure S6. JA-Ile accumulation after mechanical wounding of Arabidopsis leaves.**
The leaves of Col-0 and *bxl4* mutant lines were mechanically wounded and sampled at 0 and 2 h after wounding. Extracted levels of JA-Ile were analysed using nanoelectrospray coupled to a tandem mass spectrometer. Error bars represent SD of 3 biological replicates each performed on extracts from whole individual plants.

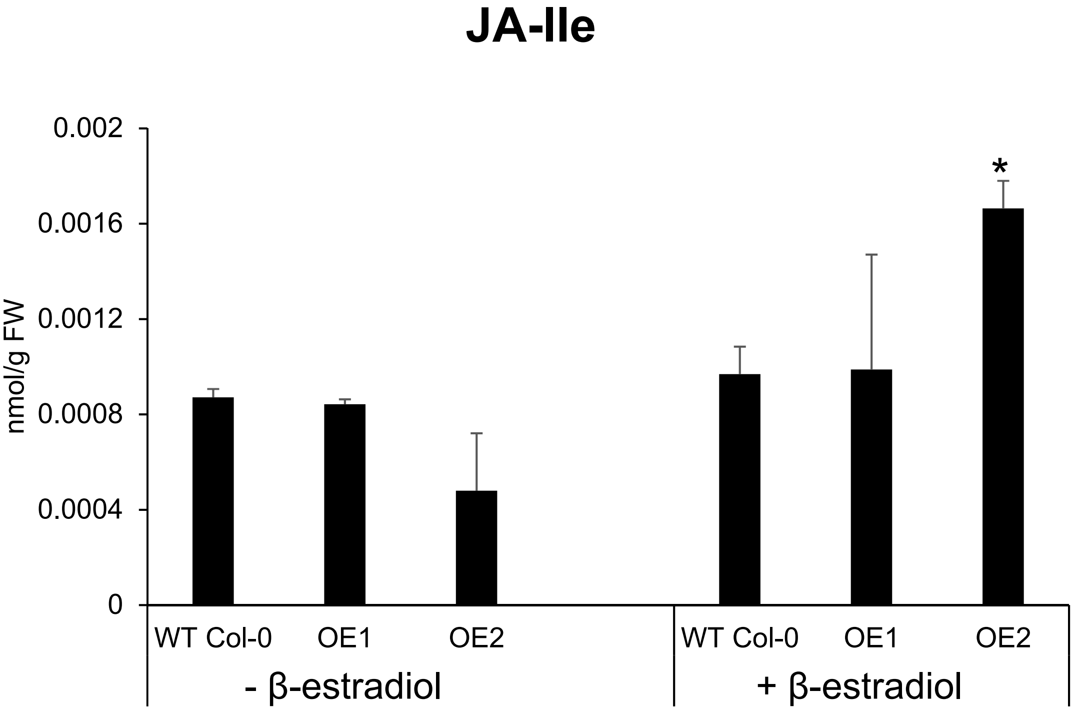


**Supplemental Figure S7. Induction of *BXL4* induces a slight accumulation of JA-Ile in Arabidopsis.** Extracted levels of JA-Ile after mock induction of *BXL4* and β-estradiol induction of *BXL4* in wild type Col-0 and β-estradiol inducible *BXL4* overexpression lines 1 and 2 (OE1 and OE2). Plants were induced with β-estradiol four days prior to harvesting and JA-Ile quantification using nanoelectrospray coupled to a tandem mass spectrometer. Error bars represent standard deviation of 6 biological replicates each performed on extracts from whole individual plants. Statistical differences to the WT were determined (Student's *t*-test * indicates $P<0.05$).

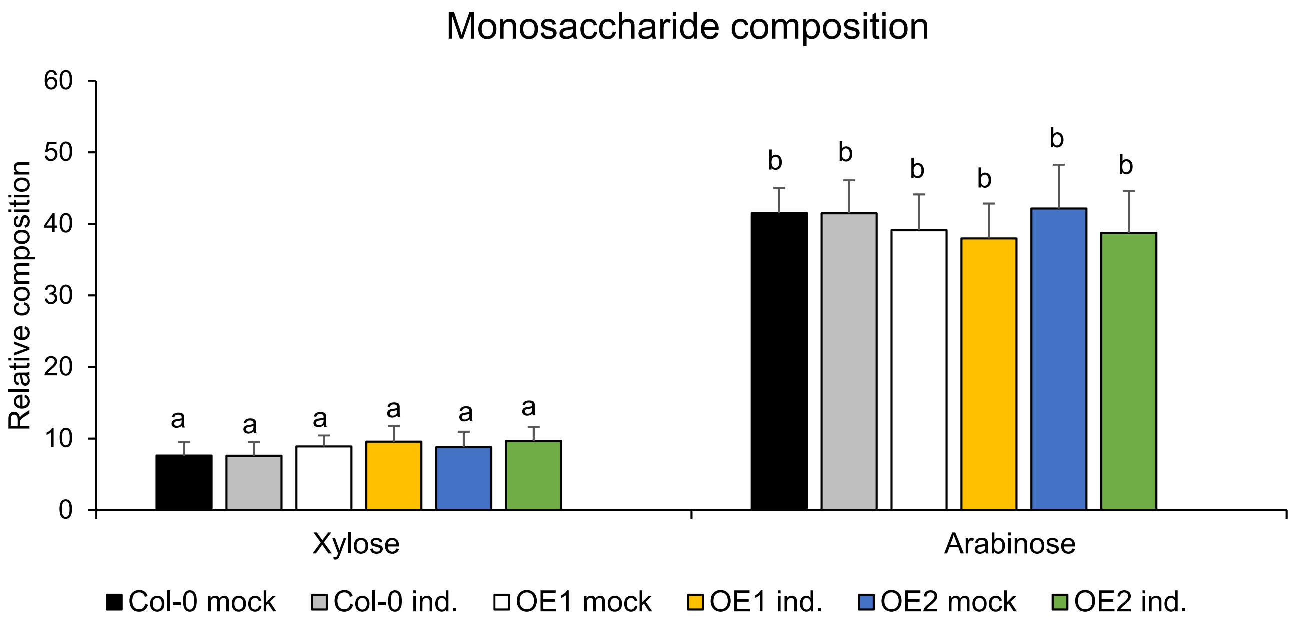


**Supplemental Figure S8. Monosaccharide composition of pectin extracted from leaf alcohol insoluble residue.**

Monosaccharide composition of water extracted pectin from Arabidopsis leaves of wild type Col-0, and β-estradiol inducible overexpression lines 1 and 2 (OE1 and OE2). The monosaccharides were normalised to total sugars. Extracted pectin was analysed by GC-MS. n=3 biological replicates each performed on extracts from whole individual plants. Error bars show SD; statistical analysis was done by one-way ANOVA with Tukey's post-hoc test. Xylose and arabinose were analysed independently of each other; different letters indicate significant differences with $p<0.05$.

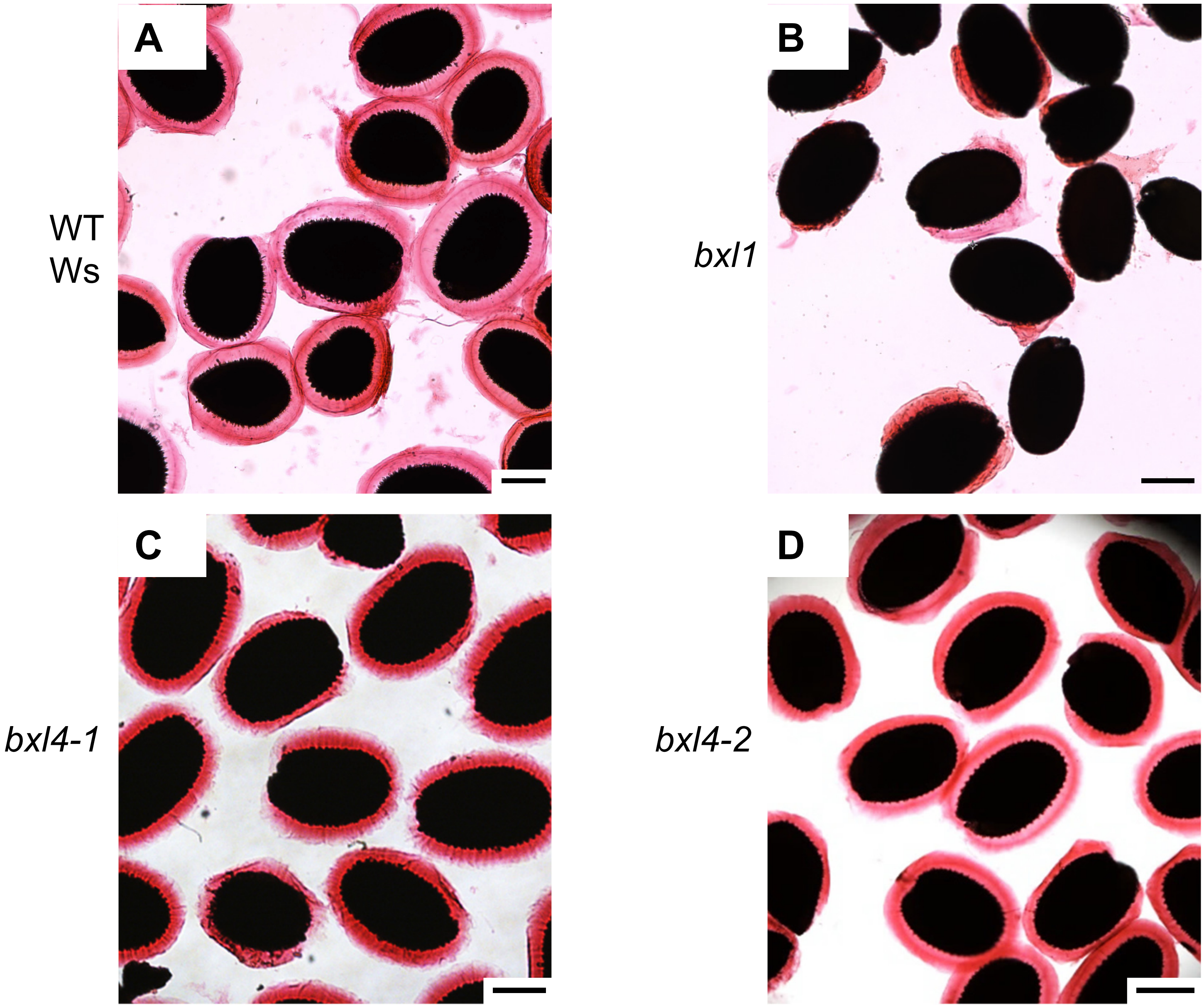


**Supplemental Figure S9. *bxl4* mutants show wild type-like extrusion of mucilage.**
The extrusion of the ruthenium red-stained mucilage from Ws, *bxl1*, *bxl4-1* and *bxl4-2* (A, B, C and D respectively). Scale bars, 200 µm. Images can be compared to wild type and *bxl1* seeds shown in figure 8, as images derive from the same experiment. Images are representative for at least 5 images per line.

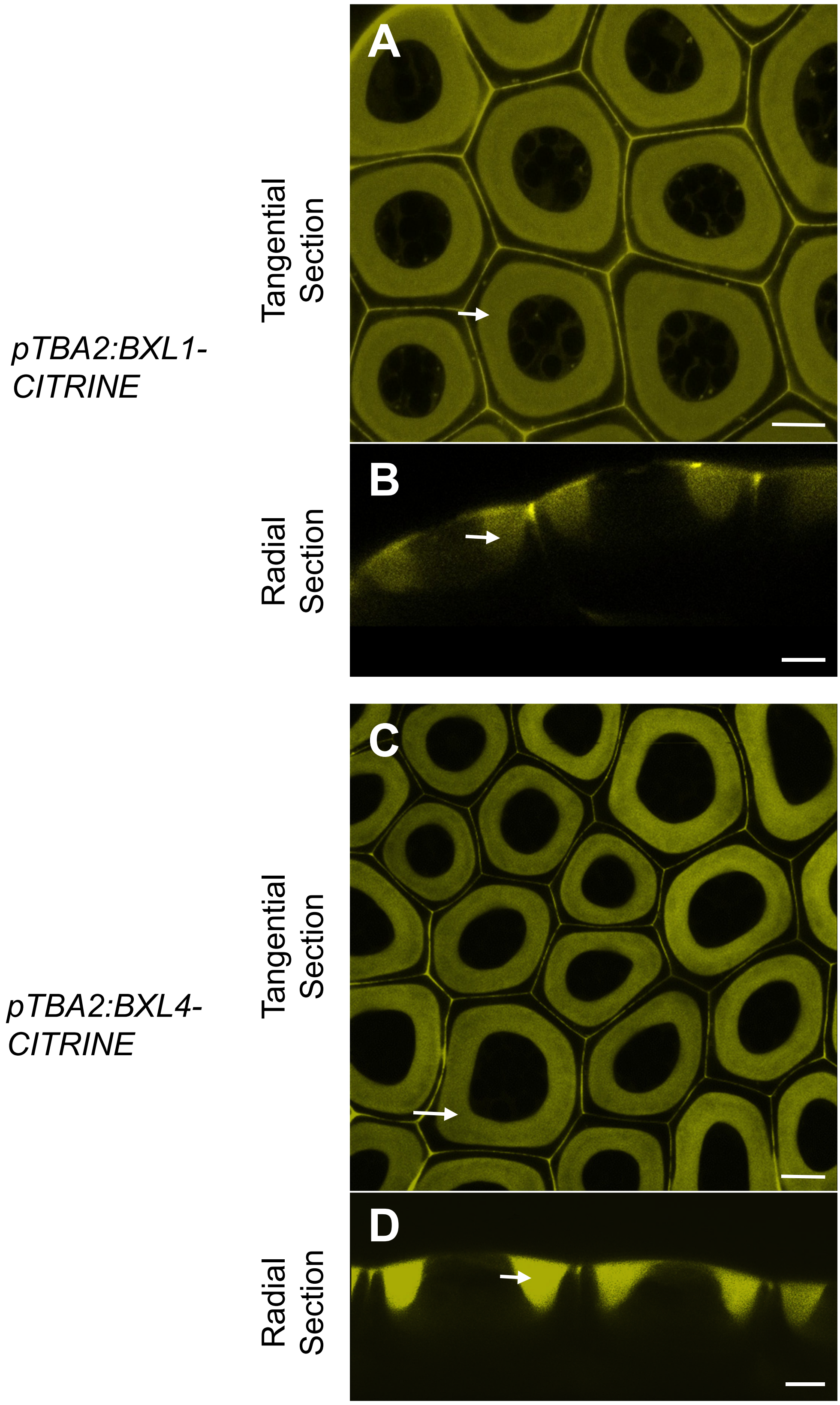


**Supplemental Figure S10. BXL1-CITRINE and BXL4-CITRINE localize to the apoplast in Arabidopsis *bxl1* seed coat epidermal cells.**

A, *BXL1-CITRINE* stably expressed under control of the TBA2 promoter localizes to the apoplast (deposited predominantly in the mucilage pocket) of Arabidopsis *bxl1* seed coat epidermal cells at 7 days post anthesis. Single plane images of the seed coats were obtained by confocal microscopy. The apopolast of seed coat epidermal cells appears like a doughnut ring (arrow) that surrounds the cytoplasm when imaged tangentially. B, visualisation of the seed coat epidermal cells radially shows the apoplast as two pockets in the apical corners of the cells (arrow). *BXL4-CITRINE* expressed by the TBA2 promoter also localises to the apoplast of Arabidopsis seed coat epidermal cells (C, D). Scale bars, 10 µm. Images are representative for 30 images from 18 seed coats derived from 4 individual lines for each construct.

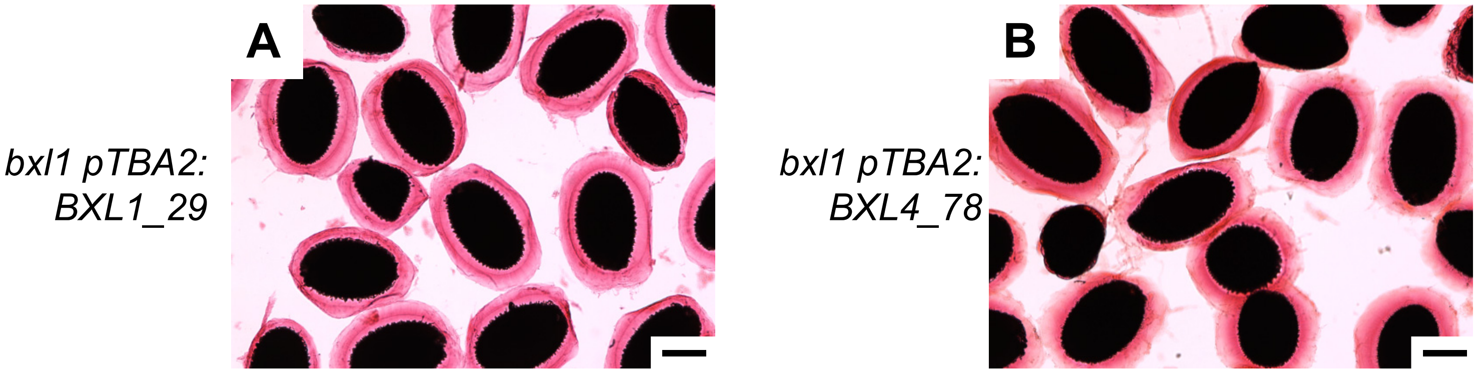


**Supplemental Figure S11. *BXL4* without a CITRINE tag complements the mucilage phenotype of *bxl1*.** Seeds of *bxl1* transformed with *pTBA2*:*BXL1* (A) and of *bxl1* transformed with *pTBA2*:*BXL4* (B) were hydrated and stained with ruthenium red. . Scale bars, 200 µm. Images can be compared to wild type and *bxl1* seeds shown in figure 8, as images derive from the same experiment. Images are representative for 30 images and at least 400 seed coats derived from 9 individual lines.

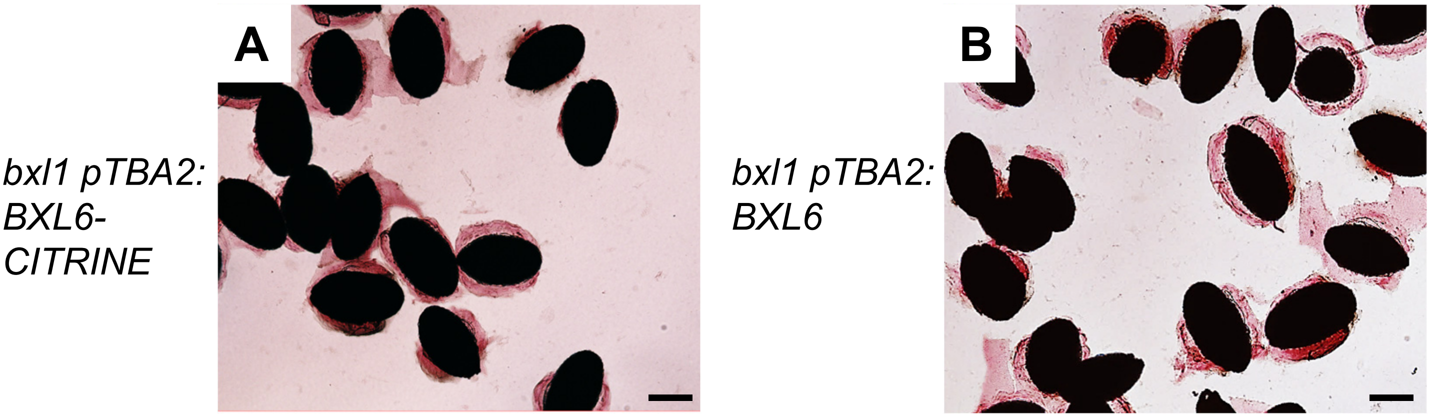


**Supplemental Figure S12. BXL6 fails to complement the mucilage phenotype of *bxl1*.**
Transgenic *bxl1* plants expressing *pTBA2*:*BXL6* fail to extrude their mucilage in a wild-type manner. Seeds of two independent *bxl1* transgenic lines expressing *pTBA2*:*BXL6-CITRINE* (A) and *pTBA2*:*BXL6* (B) were hydrated and stained with ruthenium red. Scale bars, 200 µm. Images can be compared to wild type and *bxl1* seeds shown in figure 8, as images derive from the same experiment. Images are representative for 9 images and at least 180 seed coats derived from 3 individual lines.

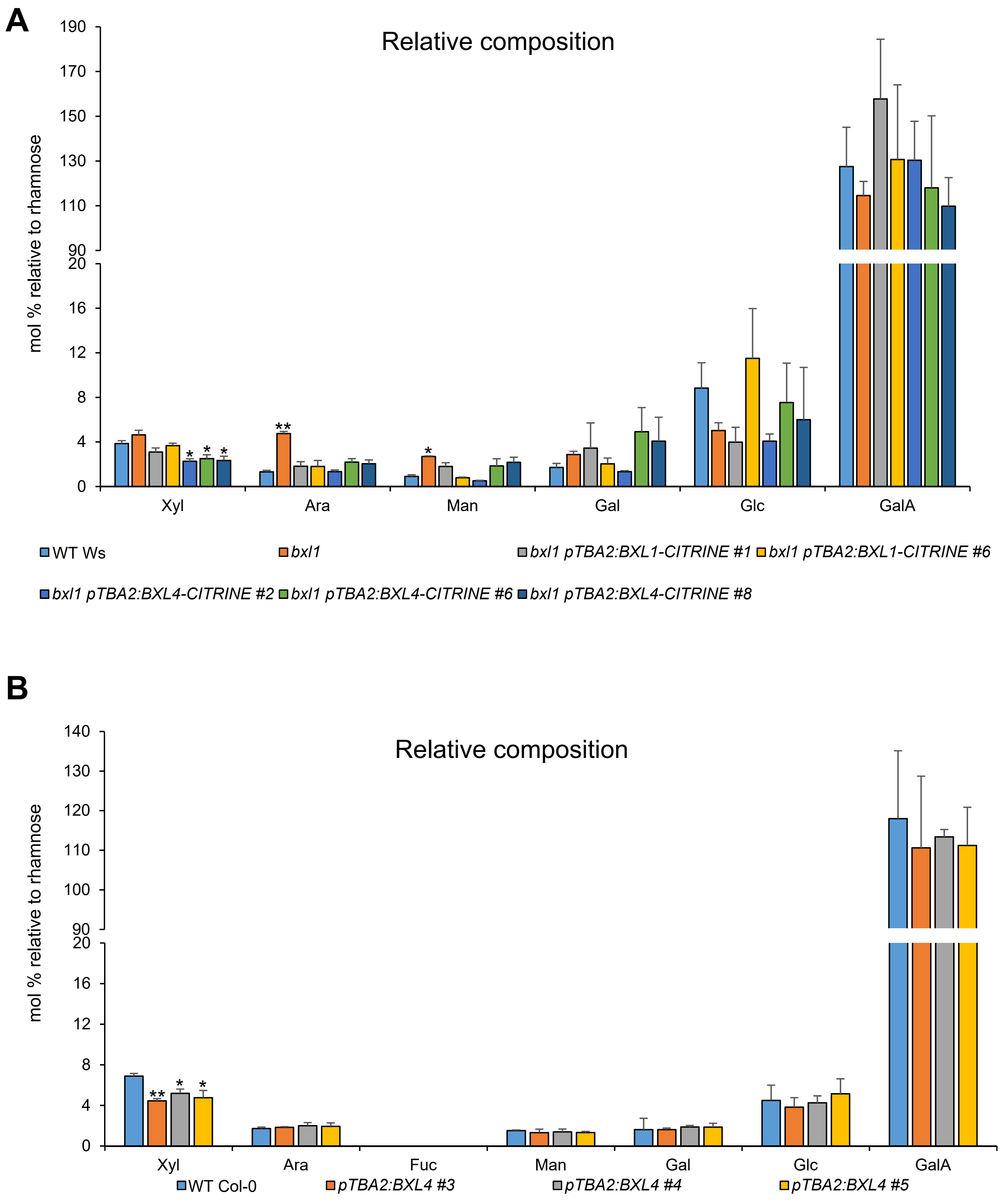


**Supplemental Figure S13. Mucilage monosaccharide composition.**
A, relative monosaccharide composition of mucilage extracted from Ws, *bxl1, bxl1* p*TBA2*:*BXL1-CITRINE* (line 1 and 6) and *bxl1* p*TBA2*:*BXL4-CITRINE* (line 2, 6 and 8) seeds. B, monosaccharide composition of mucilage extracted from wild type Col-0 and three *BXL4* overexpression lines (p*TBA2*:BXL4 line 3, 4 and 5) seeds. Monosaccharide composition was determined by GC-MS and normalised to rhamnose. n=3 biological replicates. Error bars show SD, statistical difference to the WT were determined (Student's *t*-test, * indicates p<0.05, ** p<0.01)

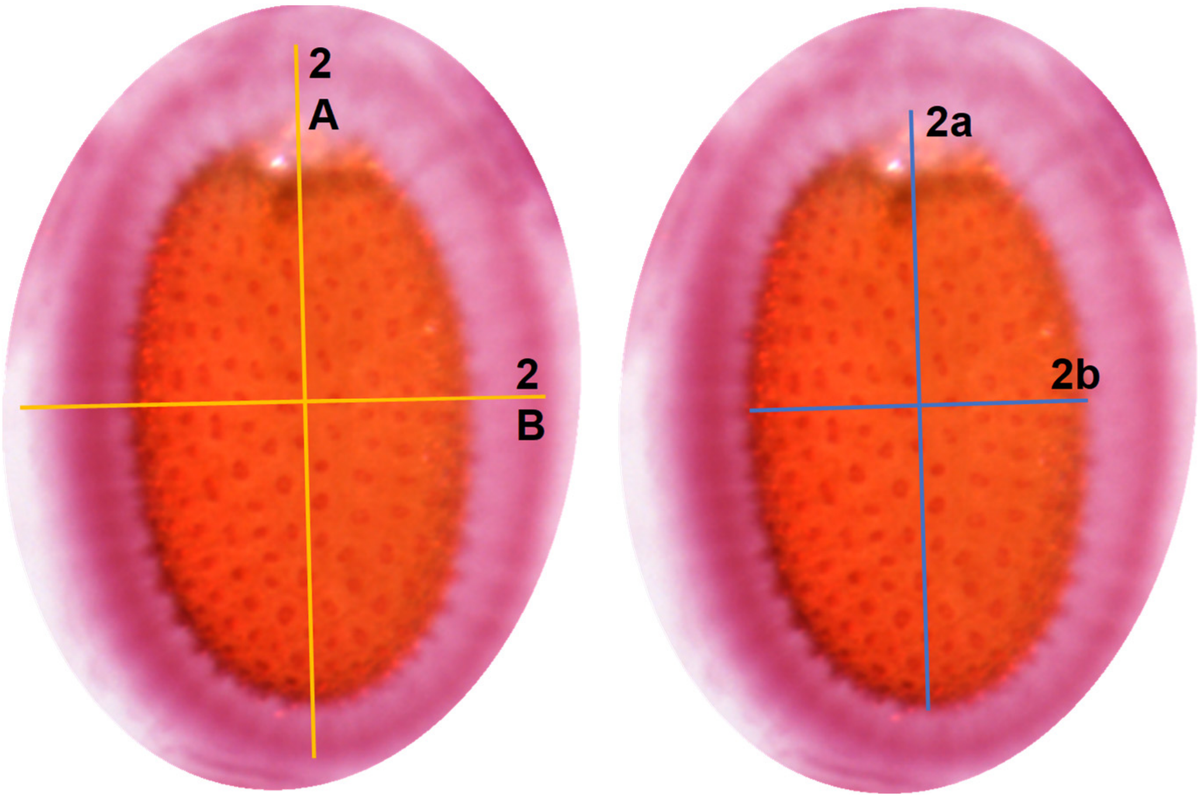


**Supplemental Figure S14 . Calculation of adherent mucilage volume.**
The shape of the seed was taken as a spheroid as described in Yu et al., (2014). The total length (2A) and width (2B) of the seed including the mucilage was measured and the volume calculated. The length of the seed alone without mucilage (2a) and the width without mucilage (2b) was measured and used to calculate the volume of the seed. The volume of the adherent mucilage was calculated by subtracting the volume of the seed alone from the total volume of the seed with mucilage using the formula: volume = 4/3 × 1/8 × length × width × depth. The width of the seed was used as the depth.
